# Enoxaparin 40 mg Versus 60 mg for Venous Thromboembolism Prophylaxis After Bariatric Surgery: A Systematic Review and Meta-Analysis of Pharmacologic and Clinical Outcomes

**DOI:** 10.1007/s11695-026-08765-4

**Published:** 2026-06-10

**Authors:** Mahmoud Albashier, Ibrahim Moqbel, Ahmad Omar Saleh, Rawan Kamal Mohammed, Mohammad Mahadin, Mohamed Hany Ezz, Mahmoud Omar Dakhly, Yahya A. Mahmoud, Hosam Elghadban

**Affiliations:** 1 Faculty of Medicine, Damietta University, Damietta, Egypt; 2 The Research Papyrus Lab, Alexandria, Egypt; 3 Faculty of Medicine, Cairo University, Cairo, Egypt; 4 The University of Jordan, Amman, Jordan; 5 Prince Hamza Hospital, Amman, Jordan; 6 Faculty of Medicine, Tanta University, Tanta, Egypt; 7 Faculty of Medicine, Minia University, Minya, Egypt; 8 Faculty of Medicine, Al-Azhar University-Gaza, Gaza, Palestinian Territory; 9 Faculty of Medicine, Mansoura University, Al Mansurah, Egypt

**Keywords:** Bariatric surgery, Enoxaparin, Venous thromboembolism, Anti-factor Xa, Thromboprophylaxis, Meta-analysis

## Abstract

**Background:**

Patients undergoing bariatric surgery face an elevated risk of venous thromboembolism (VTE), primarily due to obesity-related hypercoagulability and changes in the pharmacokinetics of anticoagulants. The standard prophylactic dosing of enoxaparin may not be adequate for this group, leading to the consideration of higher or BMI-adjusted dosing regimens. Nevertheless, the comparative efficacy and safety of standard-dose versus higher-dose enoxaparin in the context of bariatric surgery remain unclear.

**Methods:**

A systematic review and meta-analysis were performed following the PRISMA 2020 guidelines. Searches were conducted in PubMed, Embase, Scopus, Web of Science, and the Cochrane Library from their inception through September 30, 2025. The review included randomized controlled trials and cohort studies comparing enoxaparin 40 mg with 60 mg for postoperative venous thromboembolism (VTE) prophylaxis in bariatric surgery. Studies utilizing fixed-dose or BMI-tiered dosing strategies were considered eligible. The primary outcome assessed was the achievement of prophylactic anti-factor Xa levels. Secondary outcomes comprised mean anti-factor Xa levels, sub- and supra-prophylactic anti-factor Xa levels, VTE occurrence, bleeding incidents, mortality rates, overall complications, operative duration, and transfusion requirements. Random-effects meta-analyses were conducted.

**Results:**

Ten studies encompassing 1,276 patients (727 receiving 40 mg and 549 receiving 60 mg) were included. Enoxaparin 40 mg was associated with significantly lower mean anti-factor Xa levels compared with 60 mg (MD − 0.08 IU/mL; 95% CI − 0.13 to − 0.03). Patients receiving 40 mg had higher rates of sub-prophylactic anti-Xa levels (RR 1.35; 95% CI 1.03–1.77), whereas 60 mg was associated with increased supra-prophylactic levels (RR 0.25; 95% CI 0.09–0.70). Overall achievement of target prophylactic anti-Xa levels did not differ between doses, although once-daily 40 mg dosing was associated with reduced target attainment compared with once-daily 60 mg. No significant differences were observed in VTE, deep vein thrombosis, pulmonary embolism, bleeding, mortality, overall complications, operative duration, or transfusion requirements.

**Conclusions:**

Although higher enoxaparin doses improved anti-factor Xa levels, this did not translate into reductions in venous thromboembolism, bleeding, or mortality, underscoring the disconnect between pharmacokinetic markers and clinical outcomes in bariatric surgery patients.

**Registration:**

PROSPERO CRDXXXXXXXXXXX.

**Supplementary Information:**

The online version contains supplementary material available at 10.1007/s11695-026-08765-4.

## Introduction

 Venous thromboembolism (VTE) remains a major cause of postoperative morbidity and mortality worldwide. Patients with morbid obesity (BMI ≥ 40 kg/m²) face over twice the risk of developing VTE compared to those of normal weight, attributable to a confluence of limited mobility, persistent low-grade inflammation, and obesity-related metabolic comorbidities [1, 2]. Bariatric surgery is increasingly recognized worldwide as an effective and lasting solution for severe obesity [3, 4], making it crucial to enhance perioperative VTE prevention strategies in this high-risk group.

Obesity-related increases in adipose tissue, glomerular hyperfiltration, and changes in the volume of distribution can alter the pharmacokinetics of low-molecular-weight heparin (LMWH) [5, 6]. These changes could make it less effective as an anticoagulant in overweight people. LMWHs, especially enoxaparin, are commonly used for pharmacologic VTE prophylaxis due to their predictable anticoagulant effects and ease of administration [7]. However, major guidelines differ substantially in their recommendations for obese or bariatric patients, ranging from fixed dosing strategies to higher or weight-adjusted regimens [8]. Standard prophylaxis regimens usually include giving enoxaparin 40 mg under the skin once or twice a day, along with mechanical methods after surgery [9]. Nonetheless, mounting evidence suggests that fixed dosing may lead to subtherapeutic anti-factor Xa levels in individuals with a markedly elevated body mass index (BMI). This has led some medical centers to implement higher doses, such as 60 mg daily, to ensure adequate anticoagulation [10].

Existing studies evaluating various enoxaparin dosing strategies in bariatric surgery have yielded conflicting results. Some research suggests that higher doses improve anti-Xa activity, while other studies indicate no significant clinical benefit and raise concerns about the risk of bleeding [7, 10, 11]. Notably, the available literature includes two distinct types of comparative evidence: firstly, studies directly comparing fixed-dose regimens of 40 mg versus 60 mg [12, 13], and secondly, studies allocating patients to 40 mg or 60 mg based on BMI-defined stratification [14, 15], with outcomes reported separately. Since BMI-based dosing is widely used in real-world bariatric practice, both designs offer clinically relevant and complementary data for evaluating these prophylactic strategies.

Many studies are further limited by small sample sizes, variability in surgical procedures, heterogeneity in dosing protocols, and incomplete reporting of both pharmacodynamic and clinical endpoints. Importantly, improved anti-factor Xa levels do not always translate into reductions in VTE, underscoring the need to assess both laboratory and clinical outcomes concurrently. Despite widespread implementation of both dosing approaches, no prior meta-analysis has specifically compared fixed prophylactic doses of 40 mg versus 60 mg enoxaparin in bariatric surgery [16].

This study compares the effectiveness and safety of 40 mg versus 60 mg of enoxaparin for VTE prophylaxis in patients undergoing bariatric surgery. The primary outcome was the proportion of patients achieving the prophylactic anti-factor Xa target range, defined by most guidelines as 0.2–0.5 IU/mL [17]. Secondary outcomes included the incidence of postoperative VTE, major and minor bleeding events, mortality rates, anti-factor Xa levels, duration of surgery, and length of hospital stay. By synthesizing available evidence from both fixed-dose and BMI-stratified studies, this review aims to clarify the optimal prophylactic dose, reduce practice variability, and inform standardized perioperative protocols for this high-risk population.

## Methods

### Study Protocol and Registration

This systematic review and meta-analysis was conducted in accordance with the Preferred Reporting Items for Systematic Reviews and Meta-Analyses 2020 (PRISMA) [18] and the Cochrane Handbook on Systemic Reviews and Meta-Analyses [19]. The study protocol has been prospectively registered in the International Prospective Register of Systematic Reviews (PROSPERO) under registration number CRDXXXXXXXXXX.

### Eligibility Criteria

We included randomized studies, along with both prospective and retrospective cohort studies, that compared enoxaparin 40 mg to 60 mg for VTE prophylaxis in all types of bariatric surgery. Studies were deemed eligible if they directly compared fixed-dose regimens or if dosing was assigned based on BMI strata (for example, lower-BMI patients receiving 40 mg and higher-BMI patients receiving 60 mg), provided that outcomes between these groups were reported separately. By incorporating BMI-stratified cohorts, we were able to consider real-world dosing strategies, where higher doses are typically administered to patients with a greater BMI, while still allowing for a valid comparison of the 40 mg and 60 mg regimens.

The primary outcome was the proportion of patients who achieved prophylactic anti-factor Xa concentrations within the range of 0.2–0.5 IU/mL [17]. The secondary outcomes included Anti-Factor Xa Levels (IU/mL), Sub-Prophylactic Anti-Factor Xa Levels, Supra-Prophylactic Anti-Factor Xa Levels, Venous Thromboembolism (VTE), Bleeding Outcomes, Mortality, Overall Complications, Operative Duration, and transfusion requirements.

The review excluded case series, case reports, and review articles. Additional exclusion criteria included studies involving pediatric populations, those utilizing historical controls, conference abstracts that lacked complete data, and articles not published in English.

### Information Sources

A systematic and exhaustive literature search was undertaken using PubMed, Scopus, Embase, Web of Science, and the Cochrane Central Register of Controlled Trials (CENTRAL), encompassing all available records from database inception through September 30, 2025. To ensure comprehensive coverage, we also manually screened the reference lists of all eligible articles and pertinent review papers, and reviewed completed studies listed on ClinicalTrials.gov. The search was restricted to articles published in the English language. This decision was made to include only peer-reviewed publications with accessible full texts, ensuring methodological consistency and feasibility of appraisal and data synthesis.

## Search Strategy

Supplementary File 1, Table S1, presents a comprehensive overview of the search strategies employed for each database. This encompasses the MeSH terms, keywords, Boolean operators, and applied filters. The search included the terms “bariatric surgery” and “enoxaparin.” Filters for human subjects were applied, and duplicates were removed utilizing EndNote 20 [20]. Records were subsequently evaluated utilizing Rayyan [21]. Two independent reviewers evaluated the titles and abstracts for relevance, with a third reviewer resolving any discrepancies. Relevant articles were retrieved in full for screening according to predefined inclusion and exclusion criteria.

### Data Extraction

Data collection was performed using a standardized extraction template developed in Microsoft Excel and pilot-tested to ensure clarity and consistency prior to formal use. The template was organized into three primary domains. The first domain captured study-level information, including study identification details, geographic setting, total sample size, type of bariatric procedure performed, diagnostic methods for venous thromboembolism (VTE) or pulmonary embolism (PE), use of mechanical thromboprophylaxis, and length of postoperative follow-up. The second domain focused on baseline participant characteristics, documenting the number of patients allocated to each treatment group as well as key demographic and clinical variables such as age, sex distribution, body mass index (BMI), presence of diabetes mellitus, prior history of VTE, and smoking status. The third domain comprised predefined efficacy and safety endpoints, as specified in the study protocol. Data extraction was conducted independently by two reviewers to minimize errors and bias. Any disagreements were resolved through discussion and consensus, with adjudication by a senior author when necessary. If extracted information was incomplete or ambiguous, a third reviewer independently reassessed the data before it was ultimately classified as unavailable.

### Definitions

VTE was defined according to the definitions used in the included studies and generally comprised deep vein thrombosis (DVT) and pulmonary embolism (PE) confirmed by standard diagnostic methods [22]. Portal venous thrombosis was not consistently reported across studies and was therefore not included as a predefined outcome or analyzed separately.

### Risk of Bias

In alignment with the study methodology, standard tools were employed for assessing bias risk. In randomized controlled trials, two reviewers performed independent evaluations of bias risk using the Cochrane Risk of Bias 2 (RoB 2) tool [23]. Five primary domains were assessed: outcome measurement, selection of reported results, deviations from intended interventions, missing outcome data, and bias related to the randomization process. For cohort studies, the Risk Of Bias In Non-Randomized Studies of Interventions (ROBINS-I) tool was utilized [24], which assessed seven essential domains: confounding bias, participant selection bias, intervention classification bias, bias arising from deviations in intended interventions, missing data bias, outcome measurement bias, and bias in the selection of reported results. The Risk-of-bias VISualization (robvis) tool was used to generate summary figures for risk of bias [25]. The senior author resolved any disagreements.

### Statistical Analysis

A statistical analysis was conducted using RStudio (version 4.5.1) [26]. Forest plots were generated using mean difference (MD) for continuous outcomes and risk ratio (RR) for dichotomous outcomes, along with their respective 95% confidence intervals (CI). A random-effect model was adopted rather than a fixed-effect model, yielding a more conservative estimate of the pooled effect and generalizable results. Leave-one-out sensitivity analysis was performed to assess the robustness of the result by exploring the contribution of each study to the pooled effect and heterogeneity. Subgroup analysis was conducted based on dosing strategy (BMI-tiered or fixed-dose) or dosing frequency (once daily (QD) or twice daily (BID)). We evaluated potential publication bias using a DOI plot with a calculation of the Luis Furuya-Kanamori (LFK) index, which quantifies asymmetry; values within ± 1 indicate no asymmetry, values between ± 1 and ± 2 suggest minor asymmetry, and > ± 2 indicate major asymmetry. Statistical heterogeneity was assessed via I2 and Cochrane Q test values, where an I2 value of < 25% indicates a low degree of heterogeneity, 25–50% indicates a moderate degree of heterogeneity, 50–75% indicates a high degree of heterogeneity, and > 75% indicates a substantial degree of heterogeneity [27, 28]. The Cochrane Q test with a p-value of < 0.05 was considered significant for heterogeneity. The random effects model was used in all analyses regardless of heterogeneity, as recent evidence suggests that it provides a more robust outcome than the alternative fixed effects models [29].

## Results

### Study Selection

**Fig. 1:**
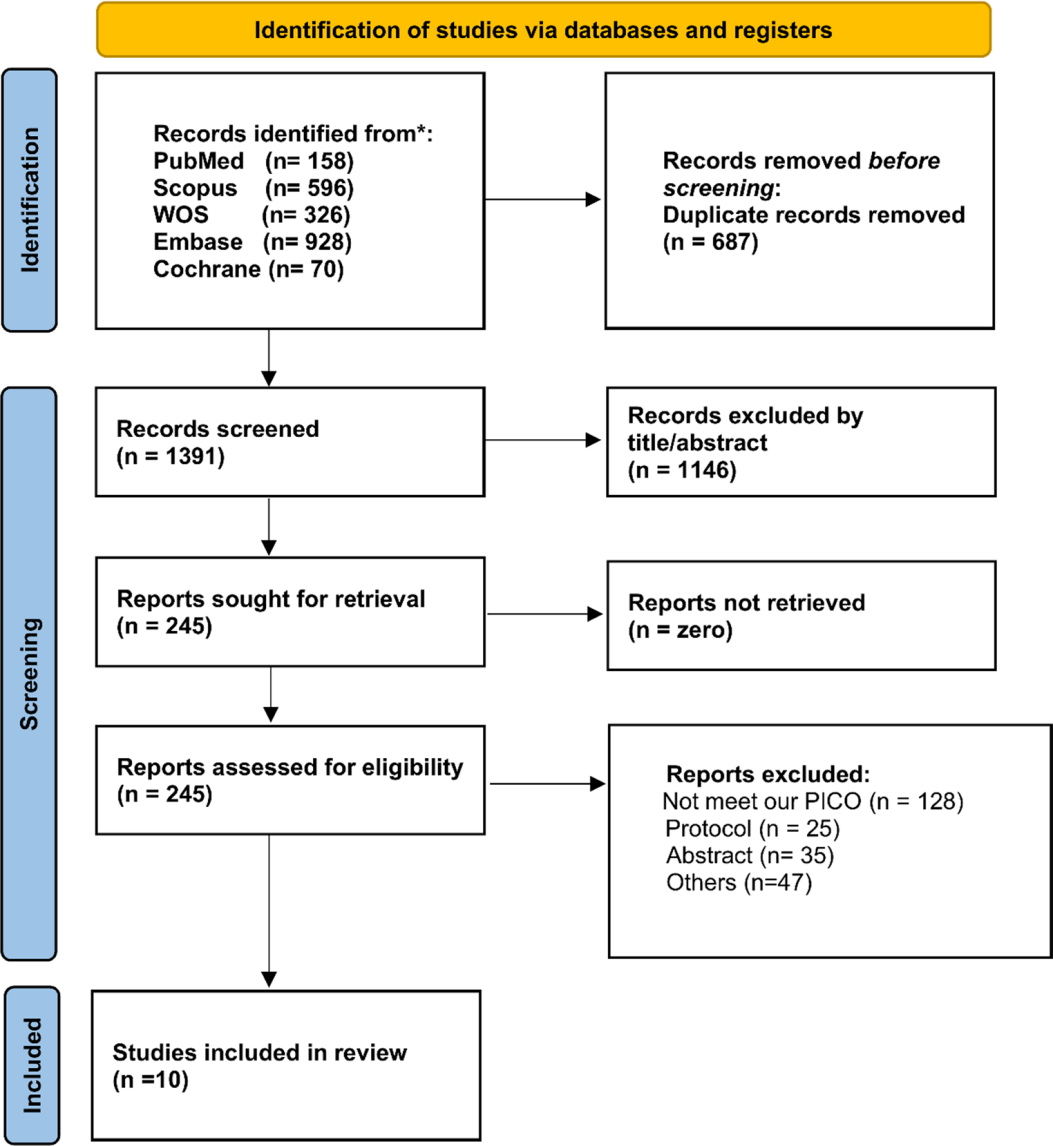
PRISMA Flow Diagram of Study Selection. Flow diagram illustrating the systematic identification, screening, eligibility assessment, and inclusion of studies comparing enoxaparin 40 mg versus 60 mg for venous thromboembolism (VTE) prophylaxis in bariatric surgery patients. A total of 2,078 records were identified through database searching, with 10 studies meeting inclusion criteria and incorporated into the quantitative meta-analysis

The systematic literature search identified a total of 2,078 records through electronic database searches. After removal of 687 duplicate entries, 1,391 unique records remained for screening. During the title and abstract review, 1,146 records were excluded for not meeting the predefined eligibility criteria. All reports sought for retrieval were successfully accessed, and no studies were excluded due to lack of full-text availability. Subsequently, 245 full-text articles were retrieved and assessed in detail for eligibility. Of these, 235 were excluded for the following reasons: failure to meet the predefined PICO criteria (*n* = 128), publication as study protocols only (*n* = 25), availability in abstract form only (*n* = 35), and other reasons (*n* = 47). Ultimately, 10 studies [12–15, 30–35] fulfilled the inclusion criteria and were incorporated into the qualitative synthesis. All included studies provided sufficient quantitative data and were therefore eligible for inclusion in the meta-analysis. The complete study selection process is illustrated in the PRISMA flow diagram **(**Fig.1**)**.

### Study Characteristics and Demographics

Across the ten included studies (2008–2025), a total of 1,276 patients undergoing bariatric surgery were analyzed, of whom 549 (43.0%) received 60 mg enoxaparin and 727 (57.0%) received 40 mg. The mean age across study arms ranged from approximately 36.8 to 48.7 years, and female patients constituted the majority in most study groups (Table 1). Study designs included randomized controlled trials and prospective and retrospective cohort studies. Surgical procedures primarily comprised Roux-en-Y gastric bypass and sleeve gastrectomy, with additional complex and revisional surgeries. Enoxaparin was administered once or twice daily using fixed-dose or BMI-tiered dosing strategies, and most studies reported mechanical prophylaxis, anti-factor Xa monitoring, and surveillance for symptomatic venous thromboembolism for up to three months postoperatively Table 2.

**Table 1:** Baseline demographic and anthropometric data from all included studies comparing 60 mg enoxaparin and 40 mg enoxaparin, which includes age, BMI, sex, and the prevalence of diabetes, smoking, hypertension, and VTE

Study ID	Arms (Doses)	Number in each group	Age (Mean ± SD) (Years)	BMI (Mean ± SD) (kg/m²)	Female *N* (%)	Diabetes *N* (%)	Hypertension *N* (%)	History of VTE *N* (%)
Connor et al., 2025 [14]	40	90	NR	NR	NR	NR	NR	NR
	60	113	NR	NR	NR	NR	NR	NR
Kongsawat et al., 2024 [33]	40	28	40.7 ± 12	45.37 ± 7.63	19(67.85)	NR	NR	0(0)
	60	28	39.5 ± 11	45.58 ± 8.85	15(53.57)	NR	NR	0(0)
Wagner et al., 2022 [15]	40	86	42.8 ± 11	48.5 ± 5.7	56(65.11)	47(54.7)	46(53.5)	2(2.4)
	60	11	36.8 ± 9	67 ± 8.5	7(63.63)	6(54.5)	5(45.5)	0(0)
Karas et al., 2021 [32]	40	70	NR	NR	NR	NR	NR	NR
	60	35	NR	NR	NR	NR	NR	NR
Stier et al., 2020 [35]	40	124	48.52	42.94 ± 6.84	101(81.45)	NR	NR	NR
	60	112	43.35	63.21 ± 10.05	65(58)	NR	NR	NR
Gelikas et al., 2017 [31]	40	31	36.94 ± 12.37	42.27 ± 0.84	21(67.74)	9(29)	6(19.4)	0(0)
	60	23	39 ± 11.04	44.15 ± 1.25	15(65.21)	4(17.4)	7(30.4)	0(0)
Steib et al., 2015 [34]	40 QD	44	39 ± 1.5	49 ± 1	33(75)	9(20.4)	19(43.2)	NR
	60	44	40 ± 1.5	48 ± 1	39(88.6)	6(13.6)	20(45.5)	NR
	40 BID	47	39.5 ± 1.7	47 ± 1	34(72.4)	13(27.6)	19(40.4)	NR
Simone et al., 2008 [13]	40	24	40.0 ± 9.8	48.8 ± 6.6	21(87.5)	NR	NR	NR
	60	16	41.0 ± 10.2	47.3 ± 6.6	15(93.7)	NR	NR	NR
Okonek et al., 2008 [30]	40	124	44.7± 10.1	44.9 ± 33.7	96(77.4)	36(29)	56(45.2)	0(0)
	60	99	44.3± 10.6	57.4 ± 66.4	72(72.7)	30(30.3)	36(36.4)	0(0)
Ojo et al., 2008 [12]	40	59	48 ± 11.1	57 ± 6	39(66.1)	NR	NR	NR
	60	68	46 ± 10.4	58 ± 8.2	26(38.2)	NR	NR	NR

*NR *Not Reported, *BMI* Body Mass Index, *VTE* Venous Thromboembolism, ***N*** Number of patients, % Percentage, Mean ± SD: Mean ± Standard Deviation, *QD* Once daily, BID: Twice daily

**Table 2:** Characteristics of included studies evaluating enoxaparin thromboprophylaxis dosing strategies in bariatric surgery patients

Study ID	Year	Study design	Country	Total sample size	Surgical procedure	Enoxaparin		VTE prophylaxis included mechanical measures	Method of (VTE / PE) ascertainment	Duration of follow-up
						Doses	Dosing Strategy			
Connor et al. [14]	2025	Retrospective Cohort	USA	203	RNYGB, LSG, Revisional	40 mg (BMI < 50), 60 mg (BMI ≥ 50)	BMI-tiered	Yes (SCDs, early ambulation)	Clinical surveillance (implied for complications)	Hospital stay + post-discharge (not specified)
Gelikas et al. [31]	2017	Prospective Cohort	Israel	54	Laparoscopic Sleeve Gastrectomy	40 mg, 60 mg	Fixed-dose	Yes (SCDs, early ambulation, IV hydration)	Anti-FXa levels; Clinical VTE surveillance	3 days post-op (for study outcomes)
Karas et al. [32]	2021	Observational Cohort	USA	105	RYGB, SG, Duodenal Switch, Revisional	40 mg (BMI < 50), 60 mg (BMI ≥ 50)	BMI-tiered	Yes (Intra-op pneumatic device, early ambulation)	Anti-FXa levels; Clinical VTE surveillance	30–90 days (for complications)
Kongsawat et al. [33]	2024	Randomized Controlled Trial	Thailand	56	Bariatric Surgery (RYGB, SG implied)	40 mg single dose, 60 mg single dose	Fixed-dose	Yes (SCDs, early ambulation)	Anti-FXa levels; Symptomatic VTE surveillance	During hospitalization
Ojo et al. [12]	2008	Retrospective Cohort	USA	127	Open Gastric Bypass	40 mg, 60 mg	Fixed-dose	Yes (Protocol included mechanical measures, not specified in detail)	Clinical surveillance for bleeding; VTE outcomes not primary (mentioned one fatal PE)	2 weeks (duration of prophylaxis) / 30 days (mortality mention)
Borkgren-Okonek et al. [30]	2008	Prospective Open Trial	USA	223	Roux-en-Y Gastric Bypass (Open & Laparoscopic)	40 mg (BMI ≤ 50), 60 mg (BMI > 50)	BMI-tiered	Yes (Pre-op UFH, IPC devices, early ambulation)	Anti-FXa levels; Clinical VTE surveillance	Up to 3 months post-op
Simone et al. [13]	2008	Prospective Cohort	USA	40	Laparoscopic Gastric Bypass, Adjustable Gastric Band	40 mg, 60 mg	Fixed-dose	NR	Anti-FXa levels; Clinical surveillance for bleeding	During hospital stay
Steib et al. [34]	2015	RCT	France	**135**	LRYGB	Group A: 4000 IU (40 mg) Group B: 6000 IU (60 mg) Group C: 2 × 4000 IU (40 mg)	Fixed-dose	Yes (Sequential compression devices ± stockings)	Doppler US (CUS) at D1, D9, D30; Clinical surveillance	1 month (post-op)
Stier et al. [35]	2020	Prospective Cohort	Germany	**236**	SG, RYGB, OAGB, Revisional	40 mg (BMI < 50), 60 mg (BMI ≥ 50)	BMI-based	Yes (Compression stockings)	Clinical surveillance for symptomatic VTE	3 months post-op
Wagner et al. [15]	2022	Prospective Cohort	Germany	**97**	SG, RYGB	Group 1 (BMI < 60): 40 mg Group 2 (BMI ≥ 60): 60 mg	BMI-based	NR	Clinical surveillance for symptomatic VTE	3 months post-op

*BMI* Body mass index (kg/m²), *CUS* Compression ultrasonography, *FXa* Factor Xa (Anti-FXa = Anti-factor Xa activity), *IPC* Intermittent pneumatic compression, *IU* International Units (40 mg enoxaparin ≈ 4000 IU), *LRYGB* Laparoscopic Roux-en-Y gastric bypass, *LSG* Laparoscopic sleeve gastrectomy, *NR* Not reported, *OAGB* One-anastomosis gastric bypass, *PE* Pulmonary embolism, *RCT* Randomized controlled trial, *RYGB* Roux-en-Y gastric bypass, *SCDs* Sequential compression devices, *SG* Sleeve gastrectomy, *UFH* Unfractionated heparin, *US* Ultrasound, *VTE* Venous thromboembolism

### Risk of Bias

**Fig. 2:**
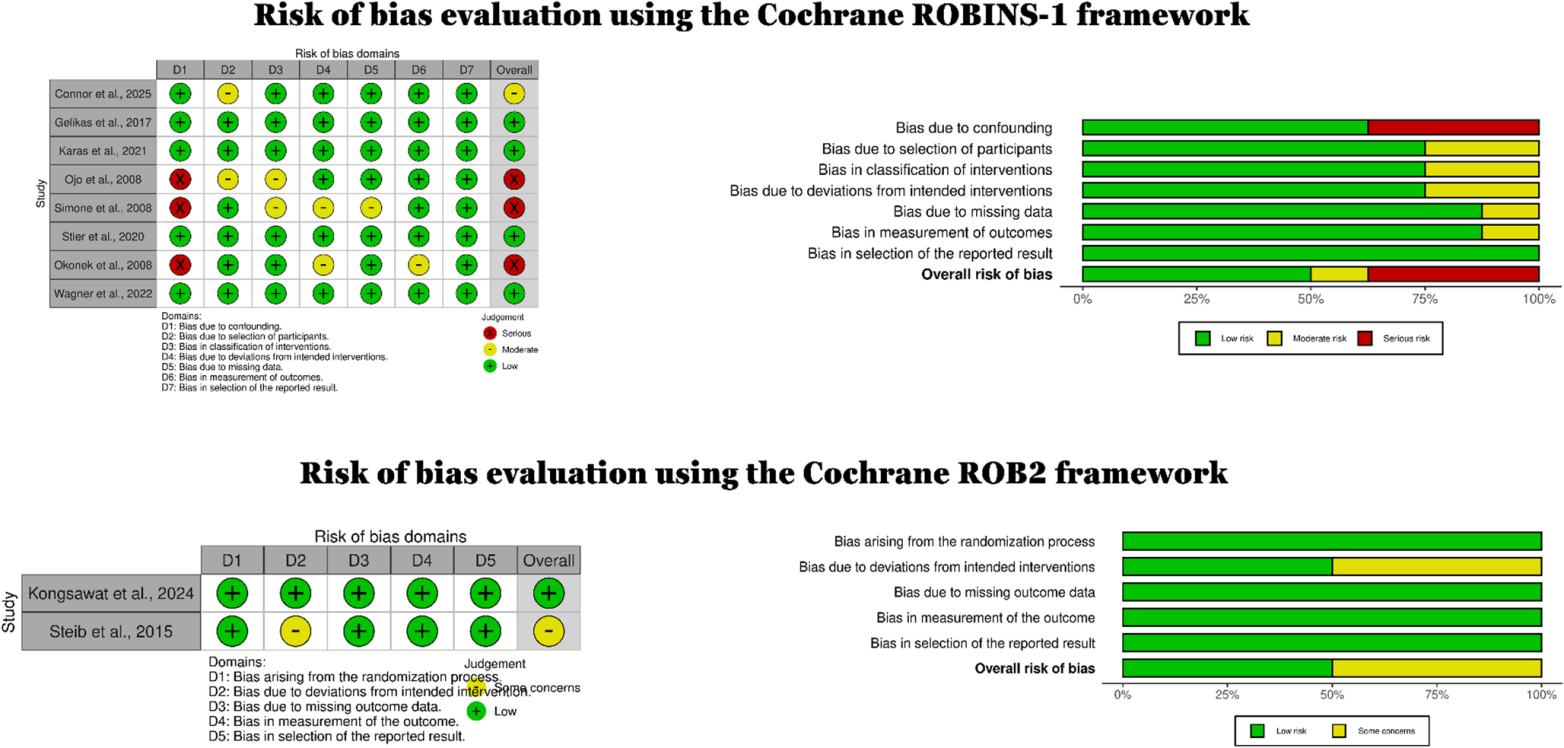
Risk of Bias Assessment of Included Studies. Risk of bias summary and traffic-light plots for included studies. Randomized controlled trials were assessed using the Cochrane Risk of Bias 2 (RoB 2) tool, and observational studies were evaluated using the ROBINS-I tool. The figure displays domain-level judgments and overall risk of bias across studies, highlighting confounding as the primary concern among non-randomized studies.

Risk of bias was assessed using the Cochrane RoB 2 tool for randomized controlled trials and the ROBINS-I tool for non-randomized studies **(**Fig. 2**).** Of the two randomized trials, one had a low risk of bias across all domains, while the other had some concerns overall, driven solely by bias due to deviations from intended interventions; both randomized studies avoided a high overall risk of bias. In contrast, the eight non-randomized studies exhibited greater variability in methodological quality: five studies were rated as having a low overall risk of bias, one study was categorized at moderate risk—mainly due to deviations from intended interventions and missing data—and three studies were identified as being at serious risk of bias, largely due to confounding factors, with further concerns related to participant selection, intervention classification, or missing outcome data. In the observational evidence, bias in the measurement of outcomes and the selection of reported results was generally low. Overall, the randomized evidence demonstrated high internal validity, while confounding emerged as the primary source of bias among the non-randomized studies; these considerations were factored into the interpretation of the findings.

## Meta-analysis

### Anti-Factor Xa Level (U/ml)

**Fig. 3:**
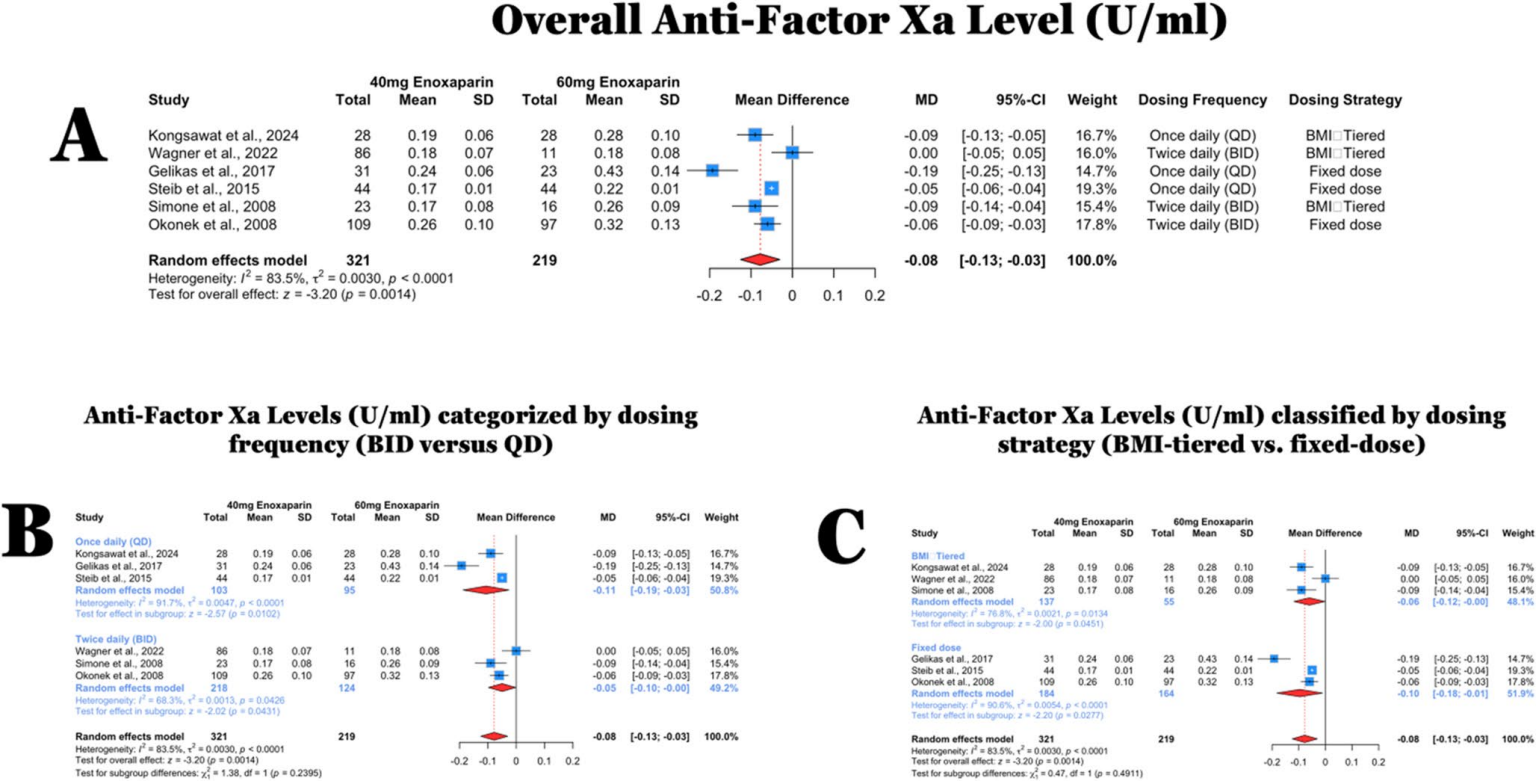
Anti-Factor Xa Levels (IU/mL).Forest plots comparing mean anti-factor Xa levels between enoxaparin 40 mg and 60 mg regimens. (**A**) Overall pooled analysis. (**B**) Subgroup analysis by dosing frequency (once daily vs. twice daily). (**C**) Subgroup analysis by dosing strategy (fixed-dose vs. BMI-tiered). Mean differences (MD) with 95% confidence intervals (CI) are shown using a random-effects model. Negative MD values favor enoxaparin 60 mg.

Enoxaparin 40 mg demonstrated a lower level of anti-factor Xa compared to enoxaparin 60 mg (MD: -0.08, 95% CI: [-0.13; -0.03], *p* = 0.0014), with substantial heterogeneity (I² = 83.5%) **(**Fig. 3A**)**. Leave-one-out sensitivity analysis revealed the result remained significant, and heterogeneity ranged from 58.2% to 86.7% after excluding each study **(Fig. ****S2****.1 A)**. Tests for subgroup analysis revealed no differences between subgroups based on dosing strategy (*p* = 0.4911) **(**Fig. 3C**)** and dosing frequency (*p* = 0.2395) **(**Fig. 3B**)**. All subgroups showed significantly lower levels of anti-factor Xa in Enoxaparin 40 mg (*p* < 0.05). The Doi plot demonstrated marked asymmetry, with an LFK index of -4.57, indicating major publication bias. This substantial deviation from symmetry suggests that the pooled effect for this outcome may be influenced by small-study effects or selective reporting **(Fig. ****S2****.1B)**.

### Sub-prophylactic anti-Xa levels

**Fig. 4:**
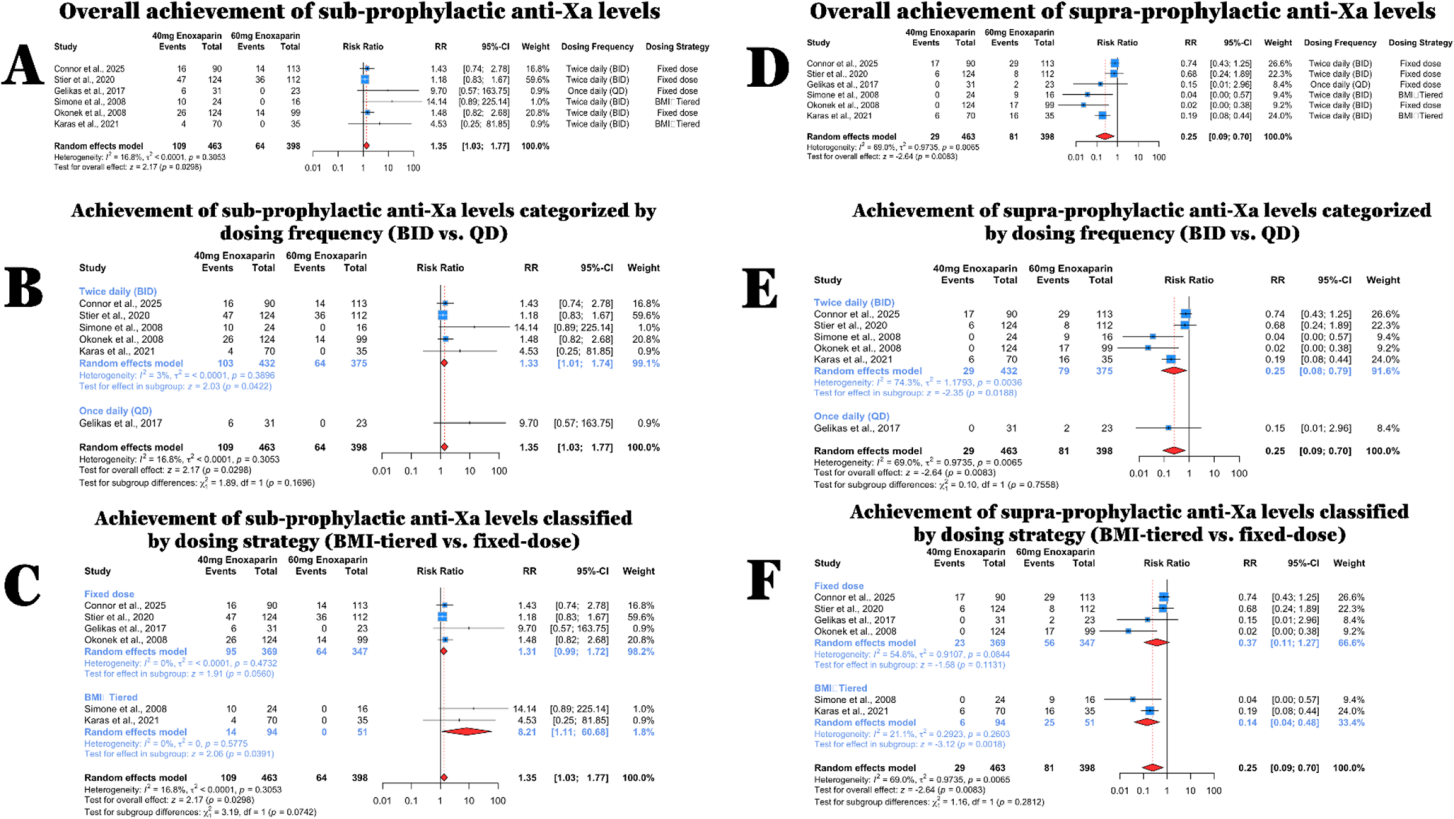
Sub-Prophylactic and Supra-Prophylactic Anti-Factor Xa Levels. Forest plots comparing rates of anti-factor Xa levels outside the prophylactic range. (**A**) Sub-prophylactic anti-Xa levels. (**B**) Subgroup analysis by dosing frequency. (**C**) Subgroup analysis by dosing strategy. (**D**) Supra-prophylactic anti-Xa levels. (**E**) Subgroup analysis by dosing frequency. (**F**) Subgroup analysis by dosing strategy. Risk ratios (RR) with 95% CI are presented using a random-effects model. RR > 1 favors enoxaparin 40 mg for sub-prophylactic outcomes and favors enoxaparin 60 mg for supra-prophylactic outcomes.

Enoxaparin 40 mg was associated with a significantly higher rate of achieving sub-prophylactic anti-Xa levels compared to enoxaparin 60 mg (RR: 1.35, 95% CI: [1.03; 1.77], *p* = 0.0298), with low heterogeneity (I² = 16.8%) **(**Fig.4A**)**. After excluding each study, the result remained robust and significant, except after excluding Connor et al. (2025) or Okonek et al. (2008), which resulted in a borderline result **(Fig. ****S2****.2 A)**. Tests for subgroup analysis showed no significant differences between subgroups based on dosing frequency (*p* = 0.1696) **(**Fig.4B**)** or dosing strategy (*p* = 0.0742) **(**Fig. 4C**)**. Both BMI-tiered doses (RR: 8.21, 95% CI: [1.11; 60.68], *p* = 0.0391; I² = 0%) **(**Fig. 4C**)** and twice-daily doses (RR: 1.33, 95% CI: [1.01; 1.74], *p* = 0.0422; I² = 3%) **(**Fig. 4B**)** resulted in a significant favoring of Enoxaparin 40 mg. The Doi plot demonstrated marked asymmetry, with an LFK index of 5.97, indicating a significant publication bias. This substantial deviation from symmetry suggests that the pooled effect for this outcome may be influenced by small-study effects or selective reporting **(Fig. ****S2****.2B)**.

### Supra-prophylactic anti-Xa levels

Enoxaparin 60 mg demonstrated a significantly higher rate of achieving supra-prophylactic anti-Xa levels compared to enoxaparin 40 mg (RR: 0.25, 95% CI: [0.09; 0.70], *p* = 0.0083), with significant heterogeneity (I² = 69.0%) **(**Fig. 4D**)**. Leave-one-out sensitivity analysis revealed the result remained significant, and heterogeneity ranged from 55% to 74.3% after excluding each study **(Fig. ****S2****.2 C)**. Tests for subgroup analysis showed no significant differences between subgroups based on dosing frequency (*p* = 0.7558) **(**Fig.4E**)** or dosing strategy (*p* = 0.2812) **(**Fig. 4F**)**. The Doi plot showed marked asymmetry, with an LFK index of -5.89, suggesting significant publication bias. This substantial deviation from symmetry suggests that the pooled effect for this outcome may be influenced by small-study effects or selective reporting **(Fig. ****S2****.2D)**.

### Achievement of Target anti-Xa levels

**Fig. 5:**
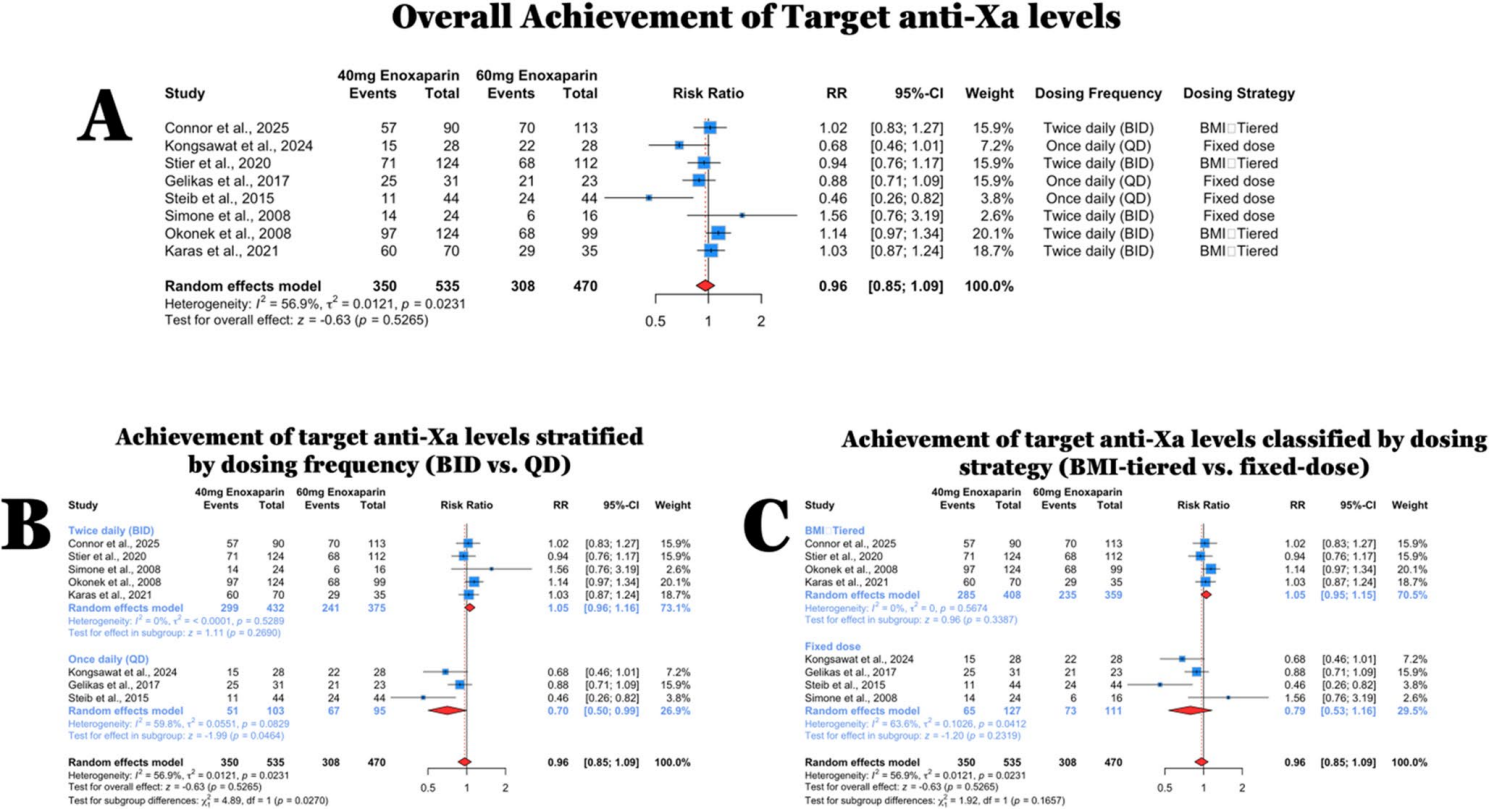
Achievement of Target Prophylactic Anti-Factor Xa Levels. Forest plots comparing the proportion of patients achieving target prophylactic anti-factor Xa levels (generally defined as 0.2–0.5 IU/mL). (**A**) Overall pooled analysis. (**B**) Subgroup analysis by dosing frequency (once daily vs twice daily). (**C**) Subgroup analysis by dosing strategy (fixed-dose vs BMI-tiered). Risk ratios (RR) with 95% CI are shown using a random-effects model

There was no significant difference between Enoxaparin 40 mg and Enoxaparin 60 mg (RR: 0.96, 95% CI: [0.85; 1.09], *p* = 0.5265), with significant heterogeneity (I² = 56.9%) **(**Fig. 5A**)**. Leave-one-out sensitivity analysis revealed that after excluding each study, the pooled effect was robust, and heterogeneity ranged from 35.3% to 62.8% **(Fig. ****S2****.3 A)**. Based on dosing frequency, the subgroup analysis revealed a significant difference between once-daily (QD) and twice-daily (BID) dosing (*p* = 0.0270). QD Enoxaparin 40 mg showed lower achievement of target AFXa compared to QD Enoxaparin 60 mg (RR: 0.70, 95% CI: [0.50; 0.99], *p* = 0.0464; I² = 59.8%). There was no difference between BID Enoxaparin 40 mg and BID Enoxaparin 60 mg (RR: 1.05, 95% CI: [0.96; 1.16], *p* = 0.269; I² = 0%) **(**Fig. 5B**)**. Based on the dosing strategy, the subgroup analysis test revealed no significant difference between BMI-tiered and fixed-dose groups (*p* = 0.1657). Both BMI-tiered and fixed-dose regimens showed no significant difference between the two doses of enoxaparin (*p* > 0.05) **(**Fig. 5C**)**. The Doi plot showed mild asymmetry, with an LFK index of **-1.72**, indicating minor publication bias. This level of asymmetry does not indicate a major distortion and is unlikely to significantly impact the pooled results **(Fig. ****S2****.3B)**.

### Venous Thromboembolism (VTE)

**Fig. 6:**
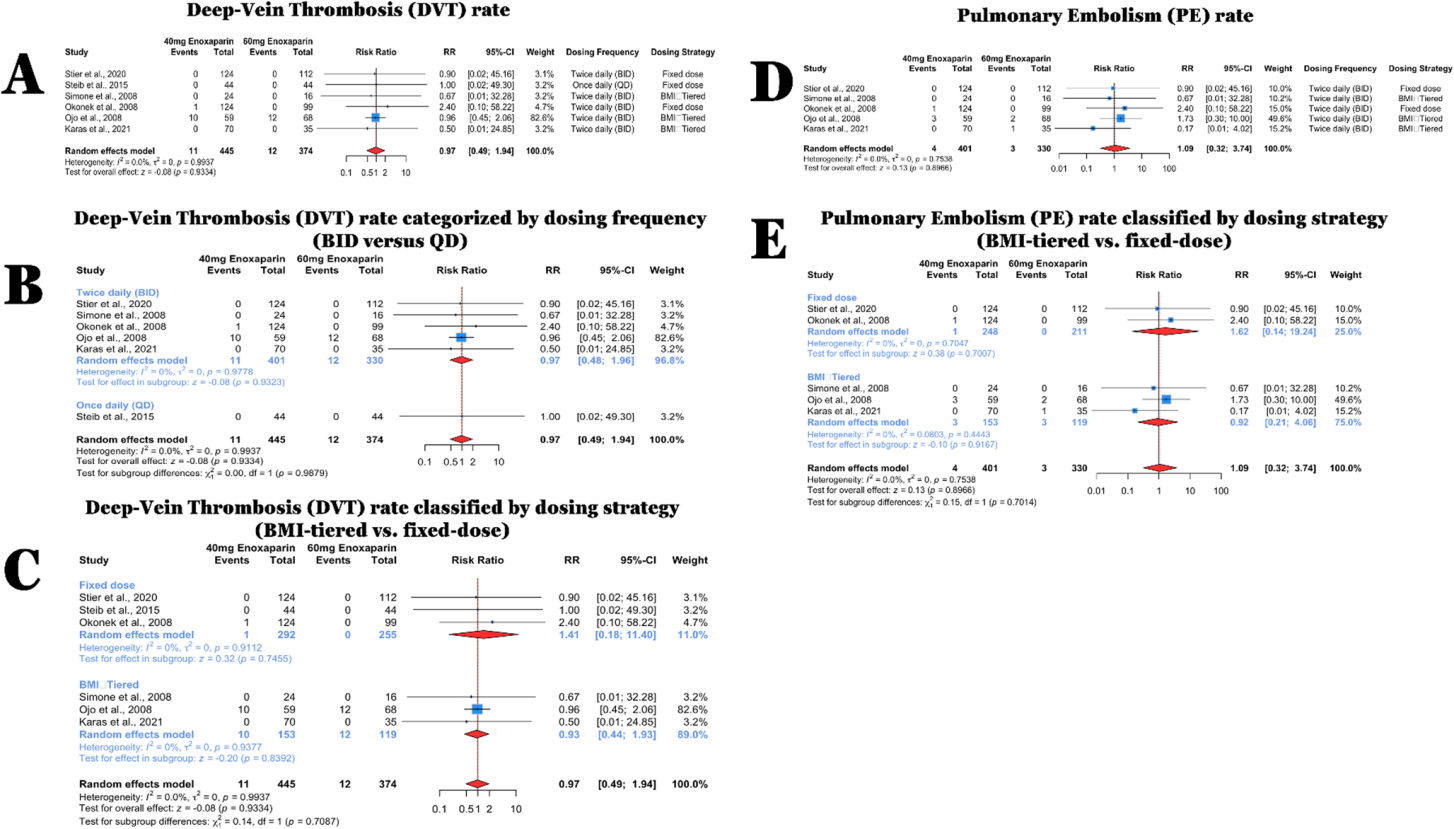
Deep Vein Thrombosis and Pulmonary Embolism. Forest plots comparing clinical thromboembolic outcomes between enoxaparin 40 mg and 60 mg. (**A**) Deep vein thrombosis (DVT). (**B**) DVT subgroup analysis by dosing frequency. (**C**) DVT subgroup analysis by dosing strategy. (**D**) Pulmonary embolism (PE). (**E**) PE subgroup analysis by dosing strategy. Risk ratios (RR) with 95% CI are presented using random-effects models

**Fig. 7:**
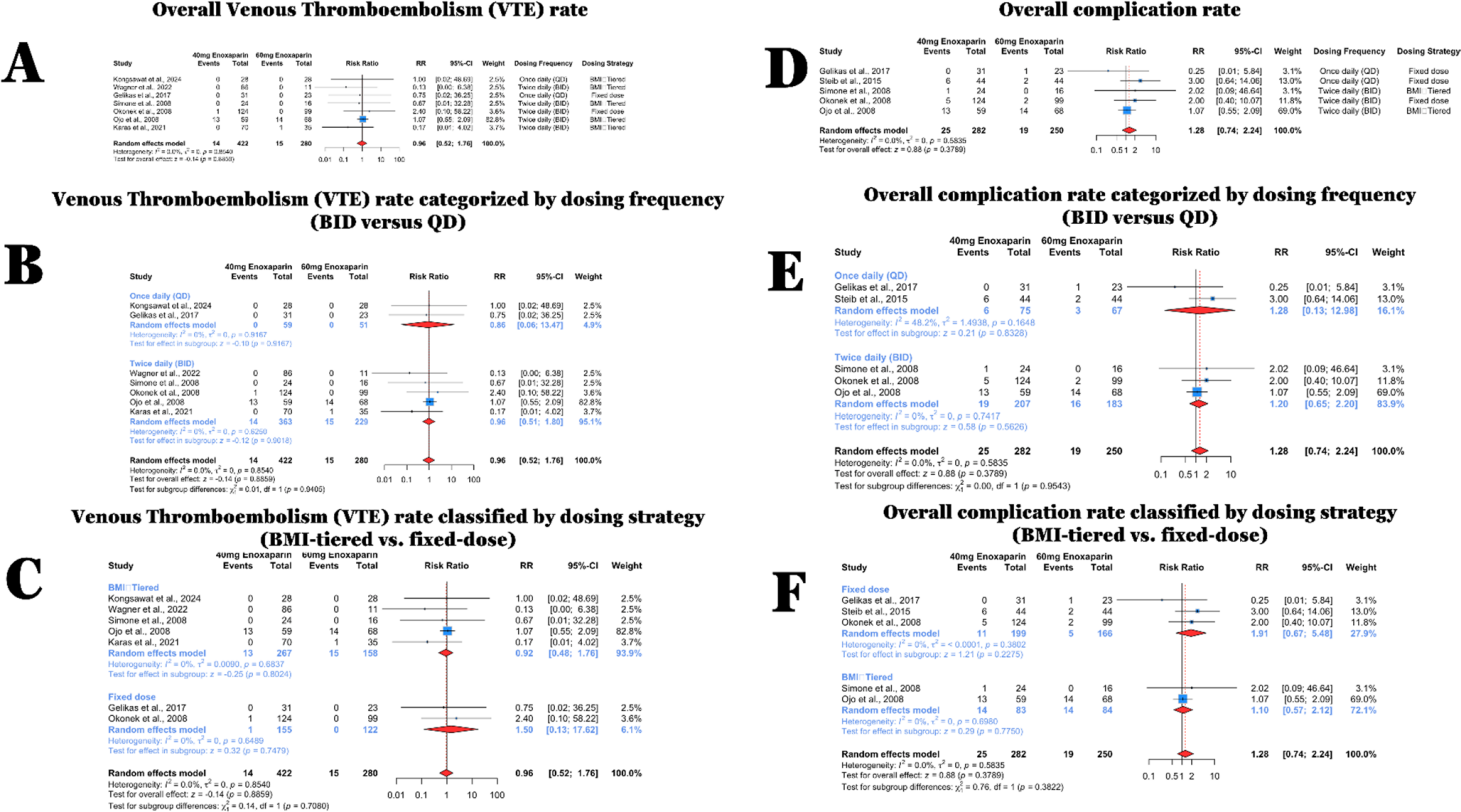
Venous Thromboembolism and Overall Complications. Forest plots summarizing postoperative clinical outcomes. (**A**) Overall venous thromboembolism (VTE). (**B**) VTE subgroup analysis by dosing frequency. (**C**) VTE subgroup analysis by dosing strategy. (**D**) Overall postoperative complication rate. (**E**) Complication subgroup analysis by dosing frequency. (**F**) Complication subgroup analysis by dosing strategy. Risk ratios (RR) with 95% CI are shown

There were no differences between Enoxaparin 40 mg and Enoxaparin 60 mg in overall VTE (RR: 0.96, 95% CI: [0.52; 1.76], *p* = 0.8859; I² = 0%) **(**Fig. 6A**)**, deep vein thrombosis (DVT) (RR: 0.97, 95% CI: [0.49; 1.94], *p* = 0.9334; I² = 0%) **(**Fig. 7A**)**, and pulmonary embolism (PE) (RR: 1.09, 95% CI: [0.32; 3.74], *p* = 0.8966; I² = 0%) **(**Fig. 7D**)**. The leave-one-out sensitivity analysis indicated that the results and heterogeneity remained consistent after excluding each study for overall VTE **(Fig. ****S2****.5 A)**, DVT **(Fig. ****S2****.4 A)**, and PE **(Fig. ****S2****.4 C)**. The subgroup analysis showed no differences between subgroups based on dosing strategy or frequency of administration. For overall VTE, dosing strategy (*p* = 0.7080) **(**Fig. 6C**)** and dosing frequency (*p* = 0.9405) **(**Fig. 6B**)** did not differ. For DVT, dosing strategy (*p* = 0.7087) **(**Fig. 7C**)** and dosing frequency (*p* = 0.9879) **(**Fig. 7B**)** also showed no differences. For PE, the dosing strategy (*p* = 0.7014) **(**Fig. 7E**)** indicated no significant differences. The Doi plot for DVT **(Fig. ****S2****.4B)** revealed no asymmetry, with an LFK index of -0.83, suggesting no significant publication bias. This finding indicates that small-study effects or selective reporting are unlikely to meaningfully influence the pooled estimate. However, the Doi plot for overall VTE **(Fig. ****S2****.5B)** and PE **(Fig. ****S2****.4D)** demonstrated marked asymmetry, with LFK indices of -4.17 and − 2.49, respectively, indicating major publication bias. This substantial deviation from symmetry suggests that the pooled effect for these outcomes may be influenced by small-study effects or selective reporting.

### Overall Complication Rate

There was no significant difference between Enoxaparin 40 mg and Enoxaparin 60 mg in the overall complication rate (RR: 1.28, 95% CI: [0.74; 2.24], *p* = 0.3789; I² = 0.0%) **(**Fig. 6D**)**. Leave-one-out sensitivity analysis revealed that the result and heterogeneity remained consistent after excluding each study **(Fig. ****S2****.5 C)**. The subgroup analysis revealed no difference between subgroups based on dosing strategy (*p* = 0.3822) **(**Fig.6 or dosing frequency (*p* = 0.9543) **(**Fig.6E**)**. The Doi plot showed mild asymmetry, with an LFK index of 1.05, indicating minor publication bias. This level of asymmetry does not suggest significant distortion and is unlikely to affect the pooled results significantly **(Fig. ****S2****.5D) Fig 7**.

### Bleeding

**Fig. 8:**
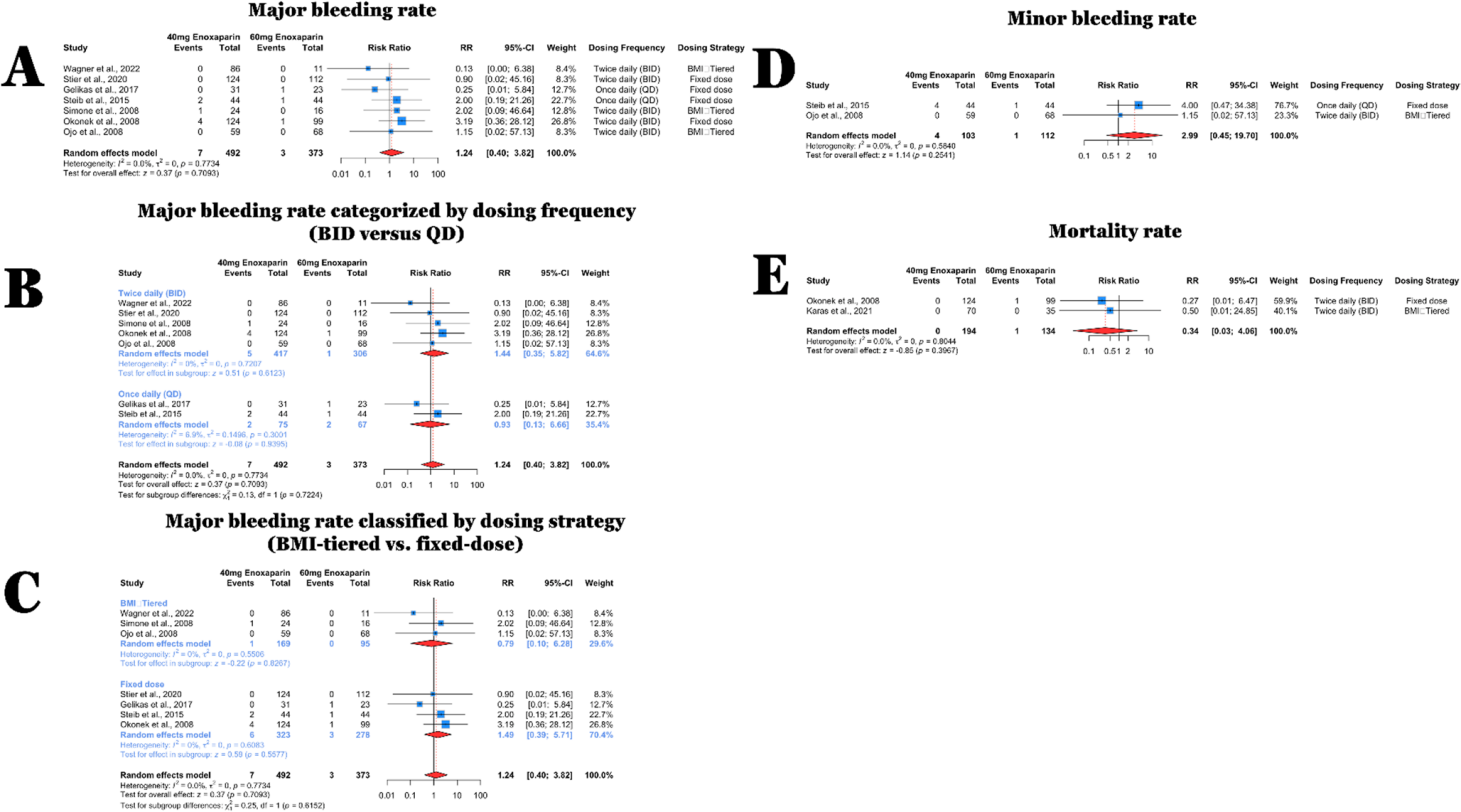
: Bleeding and Mortality Outcomes. Forest plots comparing safety outcomes between dosing regimens. (**A**) Major bleeding. (**B**) Major bleeding subgroup analysis by dosing frequency. (**C**) Major bleeding subgroup analysis by dosing strategy. (**D**) Minor bleeding. (**E**) Mortality. Risk ratios (RR) with 95% CI are presented using random-effects models

There were no significant differences between Enoxaparin 40 mg and Enoxaparin 60 mg regarding major bleeding (RR: 1.24, 95% CI: [0.40; 3.82], *p* = 0.7093; I² = 0%) **(**Fig. 8A**)** or minor bleeding (RR: 2.99, 95% CI: [0.45; 19.70], *p* = 0.2541; I² = 0%) **(**Fig. 8D**)**. The leave-one-out sensitivity analysis indicated that the results and heterogeneity remained consistent after excluding each study for major bleeding **(Fig. ****S2****.6 A)**. Subgroup analysis for major bleeding revealed no differences based on dosing strategy (*p* = 0.6152) **(**Fig. 8C**)** or dosing frequency (*p* = 0.7224) **(**Fig. 8B**)**. The Doi plot showed marked asymmetry for major bleeding, with an LFK index of − 2.65, indicating major asymmetry and suggesting the presence of potential publication bias. This implies that small-study effects or selective reporting are unlikely to significantly influence the pooled estimate **(Fig. ****S2****.6B)**.

#### Mortality

There was no significant difference in mortality rates between Enoxaparin 40 mg and Enoxaparin 60 mg (RR: 0.34, 95% CI: [0.03, 4.06]; *p* = 0.3967) with no observed heterogeneity (I² = 0%) **(**Fig. 8E**)**. Due to the limited number of studies, leave-one-out sensitivity analysis could not be reliably conducted.

### Operative duration (min) and Need for Transfusion

**Fig. 9:**
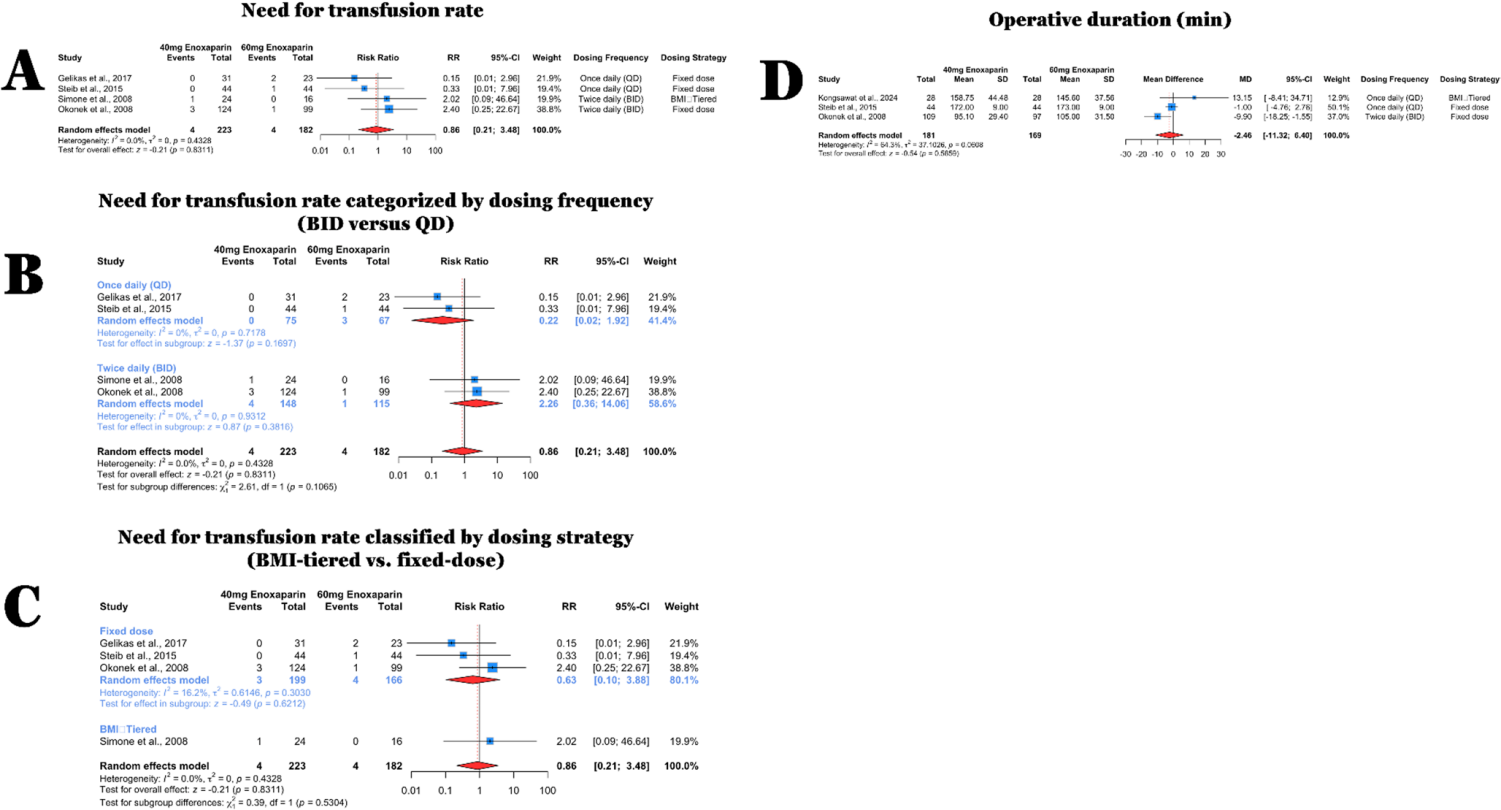
Operative Duration and Transfusion Requirements. Forest plots comparing perioperative outcomes. (**A**) Need for blood transfusion. (**B**) Transfusion subgroup analysis by dosing frequency. (**C**) Transfusion subgroup analysis by dosing strategy. (**D**) Operative duration (minutes). Risk ratios (RR) or mean differences (MD) with 95% CI are shown using random-effects models

There were no significant differences between Enoxaparin 40 mg and Enoxaparin 60 mg in the operative duration (MD: -2.46, 95% CI: [-11.32; 6.40], *p* = 0.5859; I² = 64.3%) **(**Fig. 9D**)** and in the need for transfusion (RR: 0.86, 95% CI: [0.21; 3.48], *p* = 0.8311; I² = 0.0% **(**Fig. 9A**)**. Leave-one-out sensitivity analysis revealed that the result remained nonsignificant after excluding each study for operative duration **(Fig. ****S2****.6 C)** and need for transfusion **(Fig. ****S2****.6D)**. For the need for transfusion, subgroup analysis revealed no difference between subgroups based on dosing strategy (*p* = 0.5304) **(**Fig. 9C**)** and dosing frequency (*p* = 0.1065) **(**Fig. 9B**)**. The Doi plot demonstrated marked asymmetry, with an LFK index of -2.51, indicating major publication bias. This substantial deviation from symmetry suggests that the pooled effect for this outcome may be influenced by small-study effects or selective reporting **(Fig. ****S2****.6E)**.

## Discussion

This meta-analysis shows that enoxaparin 40 mg frequently results in sub-prophylactic anti-factor Xa levels after bariatric surgery, whereas 60 mg improves pharmacologic exposure without increasing bleeding risk. However, no differences in venous thromboembolism, bleeding, mortality, or overall complications were observed. These findings highlight the limitations of fixed-dose and BMI-tiered prophylaxis and underscore the need for further research to determine whether alternative strategies, such as weight-based or anti-factor Xa–guided dosing, may improve clinical outcomes.

Enoxaparin is a low molecular weight heparin (LMWH) and an indirect anticoagulant that binds antithrombin III (serine protease inhibitor) to form a complex that irreversibly inhibits factor Xa. It has less activity against factor IIa (thrombin) than unfractionated heparin [36]. Globally, it is widely used for VTE prophylaxis across multiple surgical procedures, including hip replacement, abdominal surgery, knee replacement, and bariatric surgery [37, 38]. According to the American College of Chest Physicians, the last presurgical dose of enoxaparin should be given at least 24 h before surgery, while postoperatively, it could be readministered 12 h after surgery if indicated [22]. However, the debate continues over the optimal dosing regimen for enoxaparin in VTE prophylaxis across various surgical procedures, particularly bariatric surgery. A comprehensive assessment comparing different enoxaparin doses, considering their efficacy and associated adverse events, is therefore essential to establish updated, evidence-based guidelines for VTE prophylaxis in this patient population.

The overall pooled analysis indicated that the standard dose (40 mg) resulted in statistically significantly lower anti-Xa serum levels compared to the higher dose (60 mg). All included studies suggest this trend; however, Wagner et al. 2022 revealed no difference between the two doses [15]. This finding remained consistent across subgroup analyses by dosing frequency (once vs. twice daily) and strategy (fixed-dose vs. BMI-tiered). High between-study heterogeneity was observed, which may be attributed to variations in sample sizes, diverse surgical procedures (e.g., gastric bypass, gastric banding, sleeve gastrectomy), and differences in patient BMI ranges and sex ratios. VTE risk factors such as smoking and estrogen-containing oral contraceptive use were not controlled for between treatment groups, representing a potential source of confounding [39]. Blood sample collection timing varied across studies: some collected samples at a fixed 4-hour timepoint post-administration [33], while others used varying timeframes (ranging from 3 to 5 h in some studies versus 3 to 4 h in others) [13, 31]. This inconsistency in measurement timing may contribute to the reported heterogeneity in anti-Xa levels.

An important methodological consideration is the inclusion of BMI-tiered studies, in which enoxaparin dosing is assigned based on body mass index rather than randomized or fixed allocation. In these studies, patients receiving higher doses (e.g., 60 mg) inherently have higher BMI and potentially greater baseline thrombotic risk, introducing confounding that limits direct comparability between groups. As such, differences in anti-factor Xa levels or clinical outcomes may reflect underlying patient characteristics rather than the isolated effect of dose [40, 41]. Accordingly, while BMI-tiered studies provide valuable insight into real-world practice, they should be interpreted as confounded comparisons. In contrast, fixed-dose studies offer a more appropriate framework for evaluating the independent effect of enoxaparin dose and therefore represent the more robust basis for assessing dose–response relationships in this analysis.

These results can be explained by altered pharmacokinetics in obese patients. Obese individuals have increased plasma volume compared to normal-weight individuals. Since enoxaparin is hydrophilic and distributes primarily in plasma, the larger plasma volume in obese patients reduces drug concentration and its anti-Xa activity. Our findings are consistent with this pharmacokinetic rationale, demonstrating that the standard 40 mg dose is associated with lower anti-factor Xa levels in this population due to the increased volume of distribution [39].

The objective of achieving therapeutic anti-Xa levels is to diminish the risk of thrombosis while reducing the propensity for bleeding [42]. The target prophylactic range differed among the studies, with some specifying it as 0.2–0.4 IU/mL [14, 32, 35] and others employing a broader range of 0.18–0.44 IU/mL [13]. This difference in how thresholds are defined may have affected how patients were grouped as having target, sub-prophylactic, or supra-prophylactic levels.

However, beyond variability in target definitions, the clinical relevance of anti-factor Xa levels themselves warrants consideration. Although widely used as a pharmacodynamic marker, the relationship between anti-Xa activity and clinically meaningful outcomes such as VTE prevention remains uncertain in bariatric surgery patients [9, 41]. The commonly cited prophylactic range (0.2–0.5 IU/mL) is largely derived from non-bariatric populations and lacks robust validation in this setting. Moreover, the observed improvement in anti-Xa levels with higher enoxaparin dosing—without a corresponding reduction in thromboembolic or bleeding events—suggests that anti-Xa activity may be an imperfect surrogate for clinical efficacy [13]. These findings highlight the need for caution when interpreting anti-Xa–guided strategies and underscore the importance of prioritizing clinical outcomes when evaluating thromboprophylaxis in this population [43, 44].

Our analysis showed that enoxaparin 40 mg was linked to higher rates of sub-prophylactic anti-Xa levels (RR: 1.35, 95% CI: 1.03 to 1.77). However, the borderline statistical significance means that we should be careful about how we interpret this. This result remained consistent across subgroup analyses categorized by dosing frequency (once daily versus twice daily) and strategy (fixed-dose versus BMI-tiered). Conversely, enoxaparin 60 mg correlated with elevated rates of supra-prophylactic levels overall, aligning with results from various studies [13, 30]. This association was not significant when employing fixed-dose strategies; however, the non-significant subgroup test constrains definitive conclusions regarding strategy-specific effects. However, these findings should be interpreted with caution due to the limited sample size and number of studies.

Findings from fixed-dose studies, which provide direct head-to-head comparisons between dosing regimens, are of primary interpretive importance in assessing the independent effect of enoxaparin dose. Within this context, the pooled analysis did not show a significant difference between the two doses in terms of reaching the target prophylactic range, which is in line with several other studies [12, 13, 31, 33, 34]. However, Stier et al. 2015 suggest that the 40 mg dose has lower rates [35]. The association between enoxaparin dose and prophylactic threshold achievement remained consistent across both fixed-dose and BMI-tiered strategies in our meta-analysis. Subgroup analysis by dosing frequency revealed differential effects. Among patients receiving single-daily dosing, there was no significant difference between 40 mg and 60 mg. In contrast, among patients receiving once-daily dosing, the standard 40 mg dose showed lower odds of reaching target levels compared to 60 mg (RR: 0.70, 95% CI: 0.50 to 0.99). However, the borderline statistical significance warrants cautious interpretation.

Moderate heterogeneity was observed for this outcome. The effect and distribution of enoxaparin could be influenced by multiple patient-specific factors that were not controlled for across studies and may contribute to the reported heterogeneity. These may include renal insufficiency, hepatic function, inflammatory state, albumin levels, and edema [45, 46]. Concomitant medications should be regarded as potential confounders, as drug-drug interactions may influence the observed variations in anti-Xa levels. Connon et al. 2025 reported that patients on chronic medications such as proton pump inhibitors (PPIs) or statins for gastroesophageal reflux disease (GERD) and hyperlipidemia (HLD), respectively, were less likely to achieve target anti-Xa levels in both the 40 mg and 60 mg groups compared with other patients [14].

Individual studies assessing BMI-tiered strategies have reported variable success rates. Using a BMI threshold of 50 kg/m² (with higher doses administered to patients above this cutoff), Borkgren-Okonek et al. 2008 reported that 25.7% of 223 bariatric patients failed to achieve target anti-Xa ranges [30]. Similarly, Connor et al. 2025 found that 37% of 997 patients did not reach prophylactic levels despite BMI-adjusted dosing [14]. Kongsawat et al. 2024 and Gelikas et al. 2017 used the dose-fixed strategy and reported overall failure of 30% and 33%, respectively [31, 33]. These substantial failure rates highlight the limitations of weight-based dosing strategies in this patient population and suggest that BMI-tiered approaches may not be superior to fixed-dose regimens.

We found that VTE rates (including both DVT and PE) were slightly higher in the standard dose; however, the difference was not statistically significant (VTE, RR: 1.06, 95% CI: 0.57 to 1.94), with consistent findings across multiple studies [12, 13, 15, 30–33]. Most studies focused on anti-Xa levels, while thromboembolic events were rare across all studies, limiting the power to detect clinically meaningful differences.

Similarly, overall complication and mortality rates showed no significant differences between the two groups. Bleeding events, both minor and major, and the need for blood transfusion outcomes were also comparable. These findings are consistent with multiple previous studies in bariatric surgery cohorts, where higher prophylactic enoxaparin doses (60 mg) did not increase bleeding risks, although they increased supra-prophylactic anti-Xa levels [12, 13, 30, 31, 35].

### Strengths

This study represents, to our knowledge, the first systematic review and meta-analysis directly comparing enoxaparin 40 mg and 60 mg for VTE prophylaxis in bariatric surgery patients. We incorporated evidence from both randomized and observational studies and evaluated both pharmacokinetic (anti-factor Xa) and clinically relevant outcomes. Predefined subgroup analyses by dosing strategy and frequency further enhanced the interpretability and applicability of our findings.

### Clinical Implications

Standard enoxaparin prophylaxis (40 mg once daily) yields lower anti-factor Xa levels than higher or twice-daily regimens, but available studies have not demonstrated significant differences in clinically relevant outcomes such as VTE or bleeding. Although twice-daily dosing may improve pharmacokinetic exposure, this has not translated into clear clinical benefit and may increase treatment complexity, cost, and patient burden. Accordingly, 40 mg once daily remains a pragmatic standard approach. Dose intensification (e.g., 60 mg or twice daily) should be considered selectively in high-risk patients, including those with prior VTE, BMI ≥ 60 kg/m², known prothrombotic states, prolonged operative time, or reduced postoperative mobility. Routine anti-factor Xa monitoring is not supported by current evidence but may be reasonable in selected high-risk individuals (e.g., extreme obesity or renal impairment). Thromboprophylaxis decisions should be embedded within institutional protocols that also address mechanical measures, early mobilization, and the potential need for extended post-discharge prophylaxis. Alternative strategies, including weight-based or anti-factor Xa–guided dosing, should currently be considered investigational and reserved for selected high-risk patients until further evidence is available.

### Limitations

This study has several important limitations. First, thromboembolic and major bleeding events were infrequent, limiting statistical power to detect clinically meaningful differences and increasing the risk of type II error. Second, substantial heterogeneity was observed for certain outcomes, likely reflecting variations in surgical procedures, patient characteristics, and anti-factor Xa measurement protocols, as well as inconsistent target ranges across studies. Third, most included studies were observational, introducing potential confounding and limiting causal inference, with incomplete reporting of key variables such as comorbidities, renal function, and concomitant medications. Fourth, the predominance of single-center studies may limit generalizability, and publication bias cannot be excluded given the asymmetry observed in several Doi plots. Finally, relatively short follow-up durations may have led to underestimation of late thromboembolic or bleeding events.

### Future research

Further research is needed to address important gaps in the current evidence base. Adequately powered, multicenter randomized controlled trials focusing on clinically meaningful outcomes—such as venous thromboembolism and major bleeding—are required to determine the optimal enoxaparin dosing strategy in bariatric surgery patients. Future studies should also evaluate alternative approaches beyond fixed dosing, including weight-based and anti-factor Xa–guided regimens, and compare these with BMI-tiered strategies. In addition, longer-term follow-up is necessary to better assess the durability of prophylactic efficacy and the risk of delayed thromboembolic or bleeding events.

## Conclusion

Administration of enoxaparin 60 mg in patients undergoing bariatric surgery is associated with higher anti-factor Xa levels and a lower proportion of sub-prophylactic values compared with the standard 40 mg regimen. However, these pharmacokinetic advantages did not translate into demonstrable reductions in venous thromboembolism (VTE), bleeding events, or mortality within the included studies. Given the low incidence of clinical events and the relatively small cumulative sample size, this meta-analysis is underpowered to exclude potentially meaningful differences in these rare but important outcomes. Consequently, neither fixed 40 mg nor 60 mg regimens—administered once daily (qd) or twice daily (bid)—can be considered definitively superior based solely on currently available clinical endpoint data. Until adequately powered randomized controlled trials directly comparing weight-based and anti-factor Xa–guided prophylactic strategies are conducted, dosing decisions should be individualized. Consideration should be given to patient-specific thrombotic and bleeding risk profiles, as well as institutional protocols and resources, to optimize the balance between efficacy and safety in this high-risk population.

## Supplementary Information

Below is the link to the electronic supplementary material.

Supplementary Material 1 (DOCX 15.0 KB)

Supplementary Material 2 (DOCX 2.82 MB)

## Data Availability

This published article and its supplementary materials include all data generated or analyzed during this study.

## Abbreviations

AFXa: Anti-factor Xa
BID: Twice daily
BMI: Body mass index
CI: Confidence interval
DVT: Deep vein thrombosis
LFK: Luis Furuya-Kanamori index
LMWH: Low-molecular-weight heparin
MD: Mean difference
PE: Pulmonary embolism
PRISMA: Preferred Reporting Items for Systematic Reviews and Meta-Analyses
PROSPERO: International Prospective Register of Systematic Reviews
QD: Once daily
RCT: Randomized controlled trial
ROBINS-I: Risk Of Bias In Non-randomized Studies of Interventions
RoB 2: Cochrane Risk of Bias tool, version 2
RR: Risk ratio
SD: Standard deviation
VTE: Venous thromboembolism
