# Supplementary material for: Enoxaparin 40 mg Versus 60 mg for Venous Thromboembolism Prophylaxis After Bariatric Surgery: A Systematic Review and Meta-Analysis of Pharmacologic and Clinical Outcomes
Source: Obes Surg. 2026 Jun 10;36(7):3914–31. doi: 10.1007/s11695-026-08765-4 (PMC13323224; doi:10.1007/s11695-026-08765-4)
Supplement: Supplementary file 1 — Supplementary Material 1 (DOCX 15.0 KB) [file 11695_2026_8765_MOESM1_ESM.docx]

**Supplementary File 1**

**Table S1. Search Strategy Used for the Systematic Review (Executed on September 30, 2025)**

A systematic search was conducted across five electronic databases—PubMed, Scopus, Web of Science, Cochrane Library, and Embase—to identify studies comparing higher-dose enoxaparin (60 mg) with standard-dose enoxaparin (40 mg) for venous thromboembolism (VTE) prophylaxis in patients undergoing bariatric surgery. The search strategy employed a combination of Medical Subject Headings (MeSH) and pertinent free-text terms associated with bariatric surgery and enoxaparin.

**PubMed: 158**

("Bariatric Surgery"[Mesh] OR bariatric surgery[Title/Abstract] OR obesity surgery[Title/Abstract] OR "gastric bypass"[Title/Abstract] OR "Roux-en-Y"[Title/Abstract] OR "sleeve gastrectomy"[Title/Abstract] OR "gastric band"[Title/Abstract] OR "biliopancreatic diversion"[Title/Abstract] OR OAGB[Title/Abstract] OR "one anastomosis gastric bypass"[Title/Abstract]) AND (Enoxaparin[Mesh] OR enoxaparin[Title/Abstract] OR "low molecular weight heparin"[Title/Abstract] OR LMWH[Title/Abstract] OR clexane[Title/Abstract])

**Scopus: 596**

TITLE-ABS-KEY("Bariatric Surgery" OR bariatric surgery OR obesity surgery OR "gastric bypass" OR "Roux-en-Y" OR "sleeve gastrectomy" OR "gastric band" OR "biliopancreatic diversion" OR OAGB OR "one anastomosis gastric bypass")) AND TITLE-ABS-KEY ((enoxaparin OR enoxaparin OR "low molecular weight heparin" OR LMWH OR clexane)))

**Web of Science: 326**

TS=("Bariatric Surgery" OR bariatric surgery OR obesity surgery OR "gastric bypass" OR "Roux-en-Y" OR "sleeve gastrectomy" OR "gastric band" OR "biliopancreatic diversion" OR OAGB OR "one anastomosis gastric bypass") AND TS=(enoxaparin OR enoxaparin OR "low molecular weight heparin" OR LMWH OR clexane)

**Cochrane: 70**

("Bariatric Surgery" OR bariatric surgery OR obesity surgery OR "gastric bypass" OR "Roux-en-Y" OR "sleeve gastrectomy" OR "gastric band" OR "biliopancreatic diversion" OR OAGB OR "one anastomosis gastric bypass") AND (enoxaparin OR enoxaparin OR "low molecular weight heparin" OR LMWH OR clexane)

**Embase: 928**

('bariatric surgery'/exp OR 'bariatric surgery' OR bariatric:ti,ab OR 'obesity surgery':ti,ab OR 'gastric bypass'/exp OR 'gastric bypass':ti,ab OR 'roux en y gastric bypass':ti,ab OR 'sleeve gastrectomy'/exp OR 'sleeve gastrectomy':ti,ab OR 'gastric banding'/exp OR 'gastric band*':ti,ab OR 'biliopancreatic diversion'/exp OR 'biliopancreatic diversion':ti,ab OR OAGB:ti,ab OR 'one anastomosis gastric bypass':ti,ab) AND ('enoxaparin'/exp OR enoxaparin:ti,ab OR 'low molecular weight heparin'/exp OR 'low molecular weight heparin':ti,ab OR LMWH:ti,ab OR clexane:ti,ab)
