# Supplementary material for: Enoxaparin 40 mg Versus 60 mg for Venous Thromboembolism Prophylaxis After Bariatric Surgery: A Systematic Review and Meta-Analysis of Pharmacologic and Clinical Outcomes
Source: Obes Surg. 2026 Jun 10;36(7):3914–31. doi: 10.1007/s11695-026-08765-4 (PMC13323224; doi:10.1007/s11695-026-08765-4)
Supplement: Supplementary file 2 — Supplementary Material 2 (DOCX 2.82 MB) [file 11695_2026_8765_MOESM2_ESM.docx]

**Supplementary File 2**

**
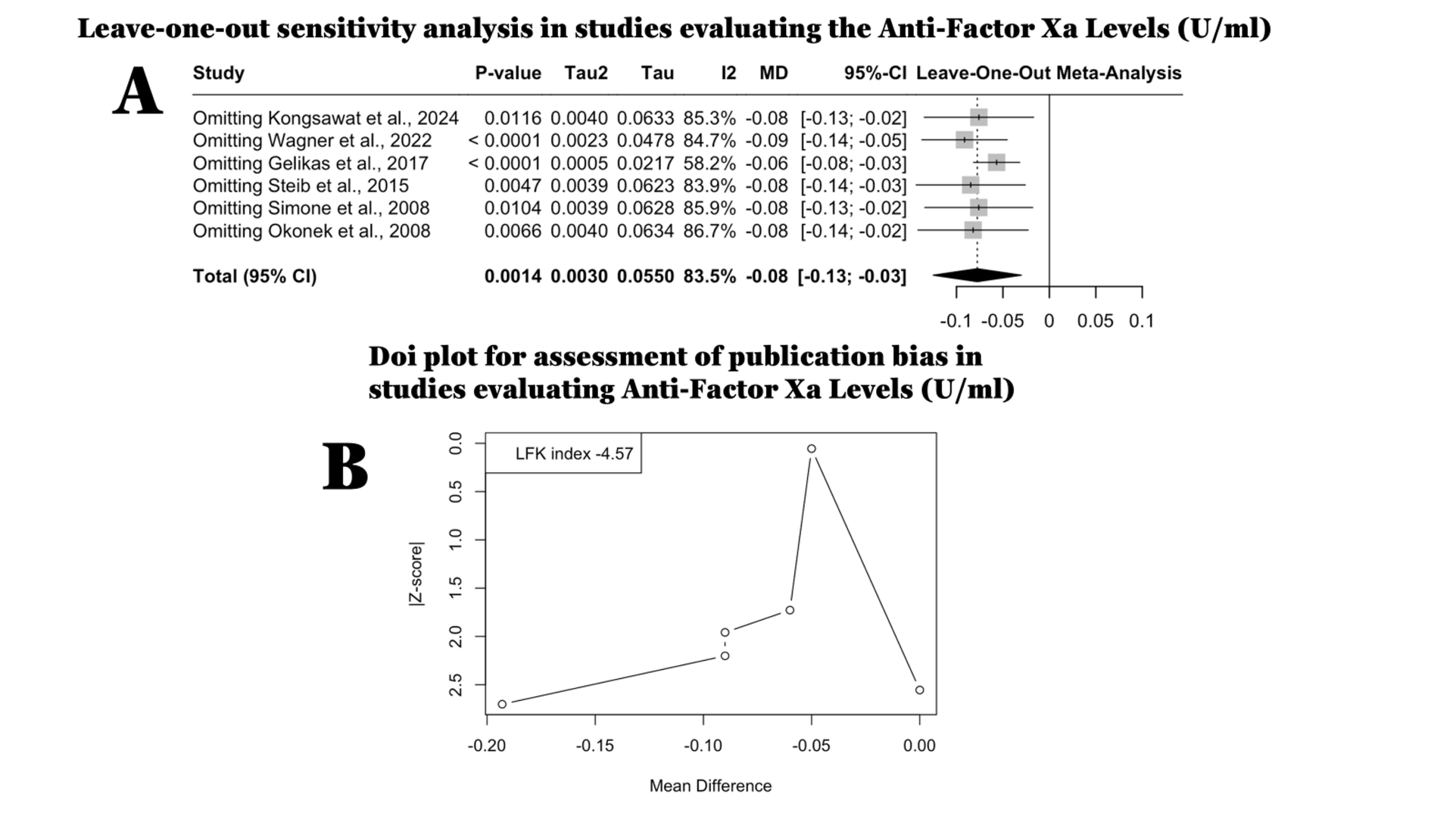
**

**(Fig. S2.1) Leave-one-out sensitivity analysis and Doi plot evaluating potential publication bias for anti-factor Xa levels.**


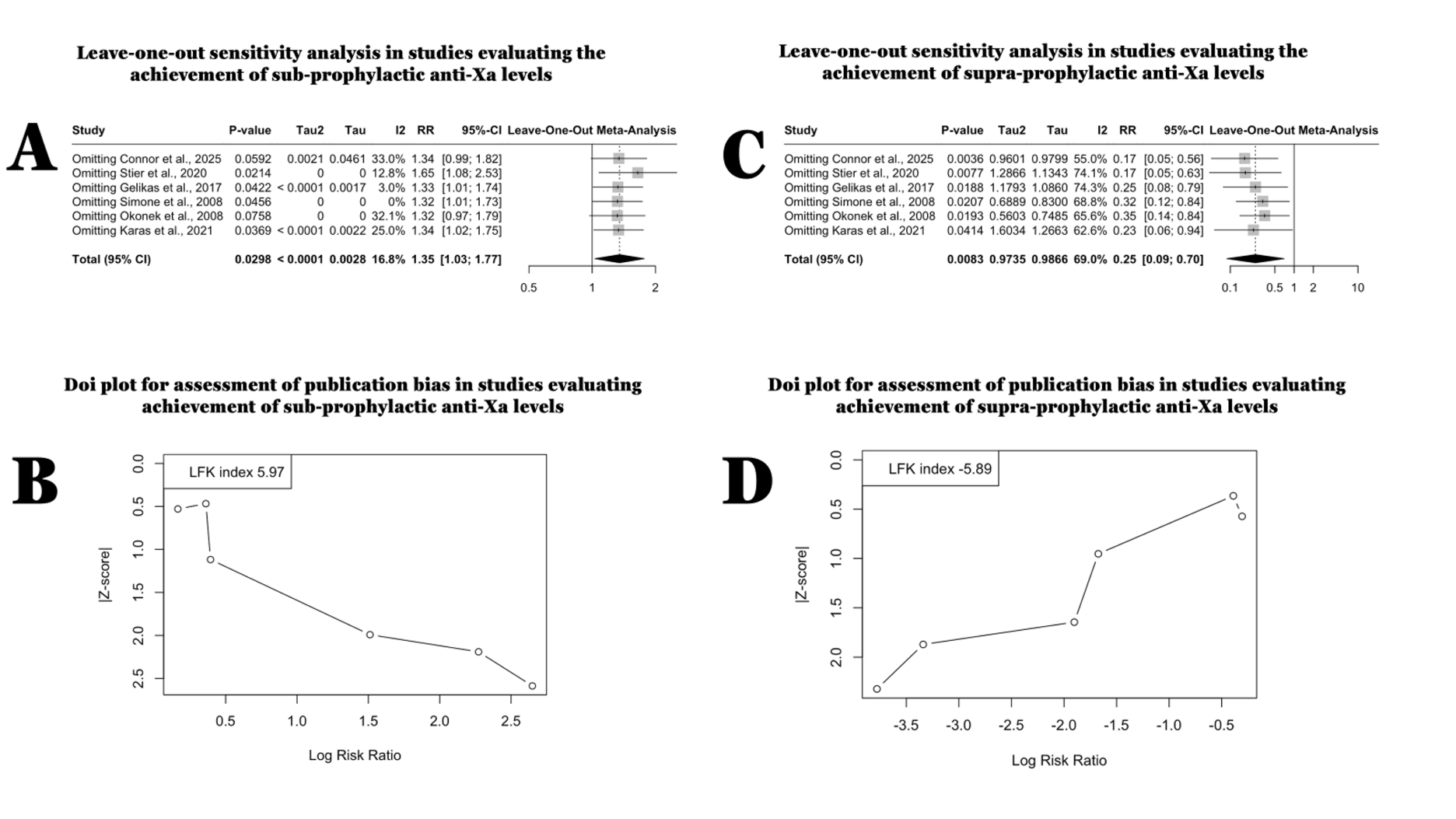


**(Fig. S2.2) Leave-one-out sensitivity analysis and Doi plot evaluating potential publication bias for sub-prophylactic and supra-prophylactic anti-Xa levels.**

**
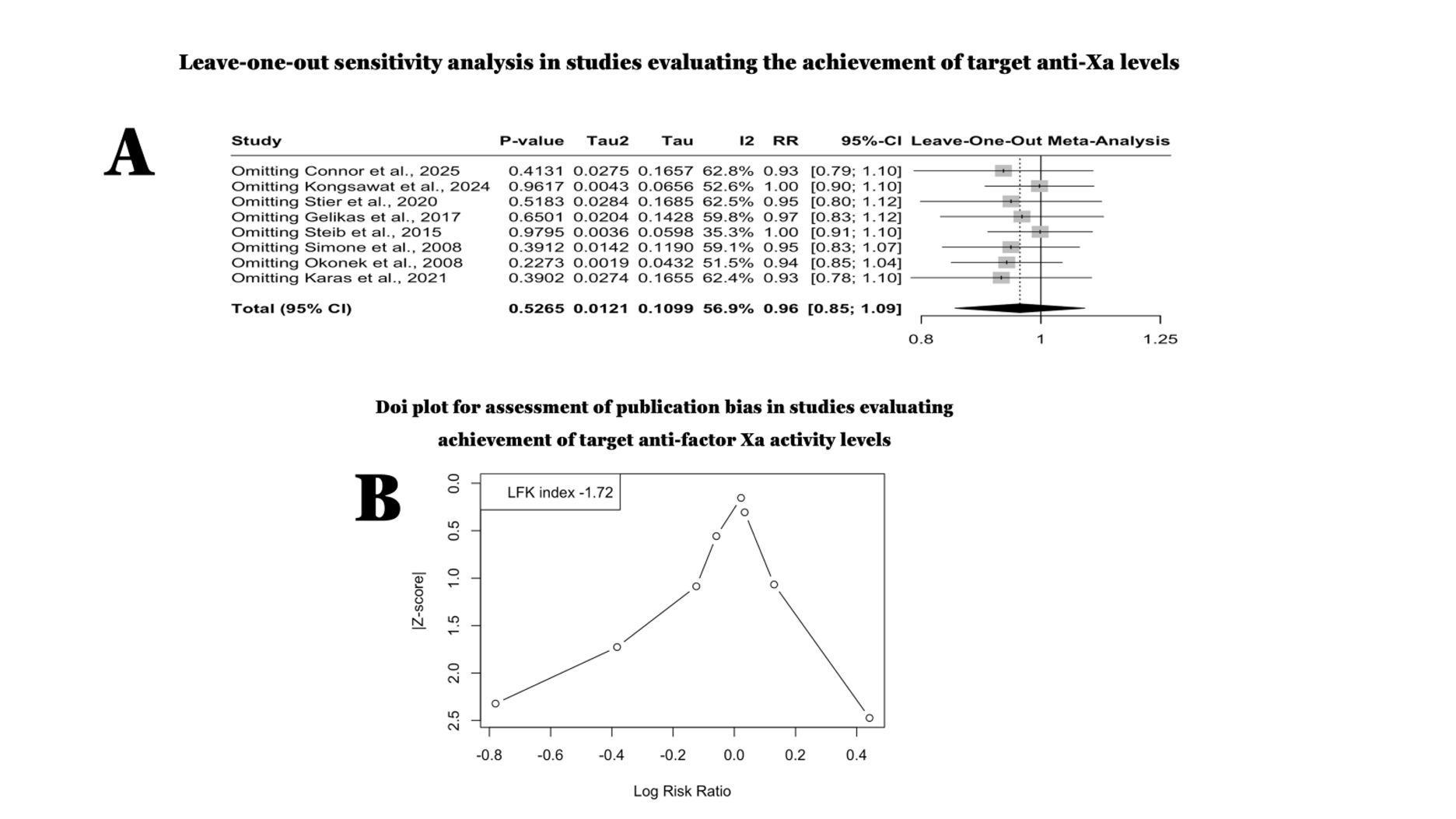
**

**(Fig. S2.3) Leave-one-out sensitivity analysis and Doi plot evaluating potential publication bias for achievement of target anti-Xa levels**

**
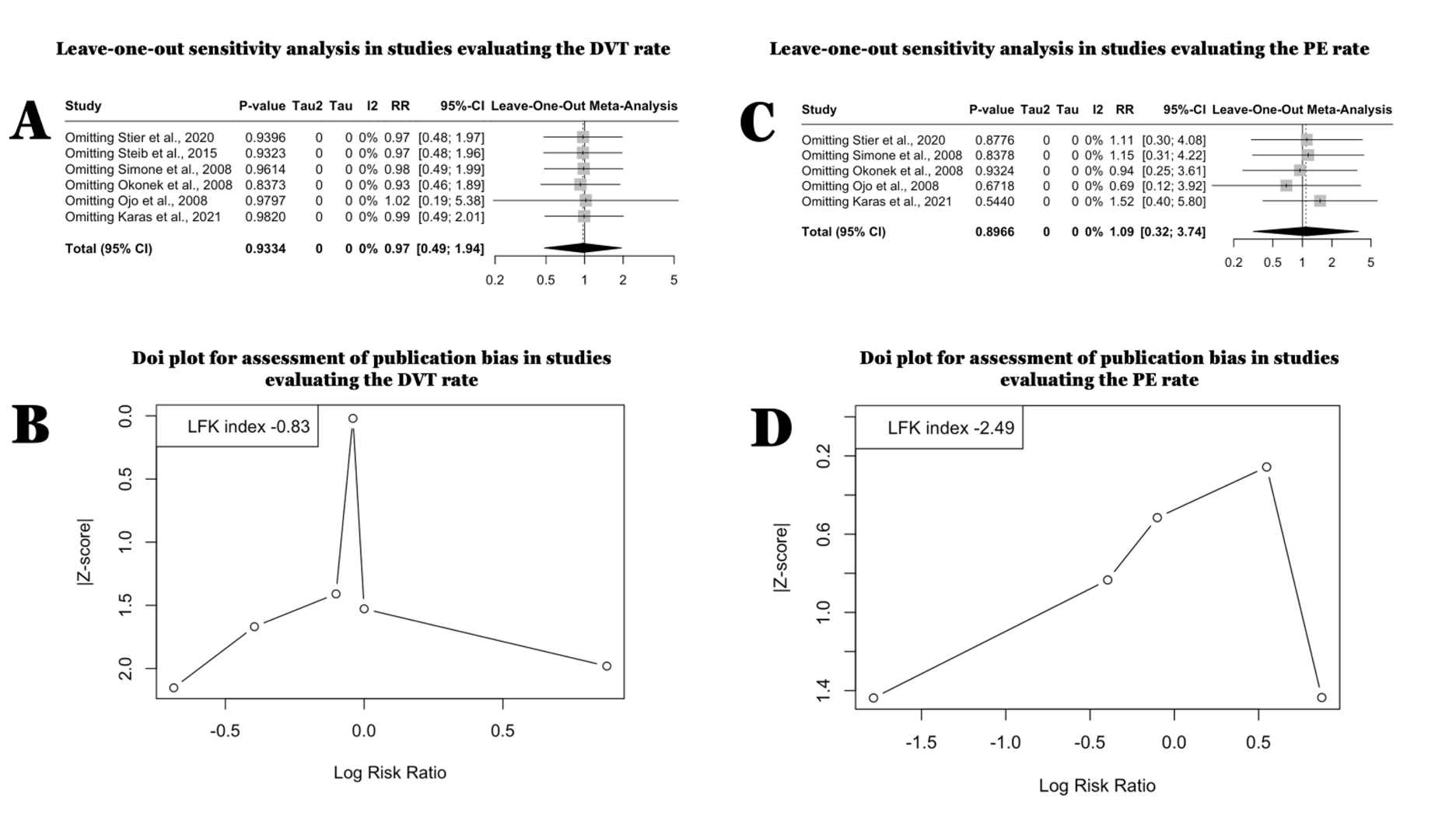
**

**(Fig. S2.4) Leave-one-out sensitivity analysis and Doi plot evaluating potential publication bias for DVT and PE rates.**

**
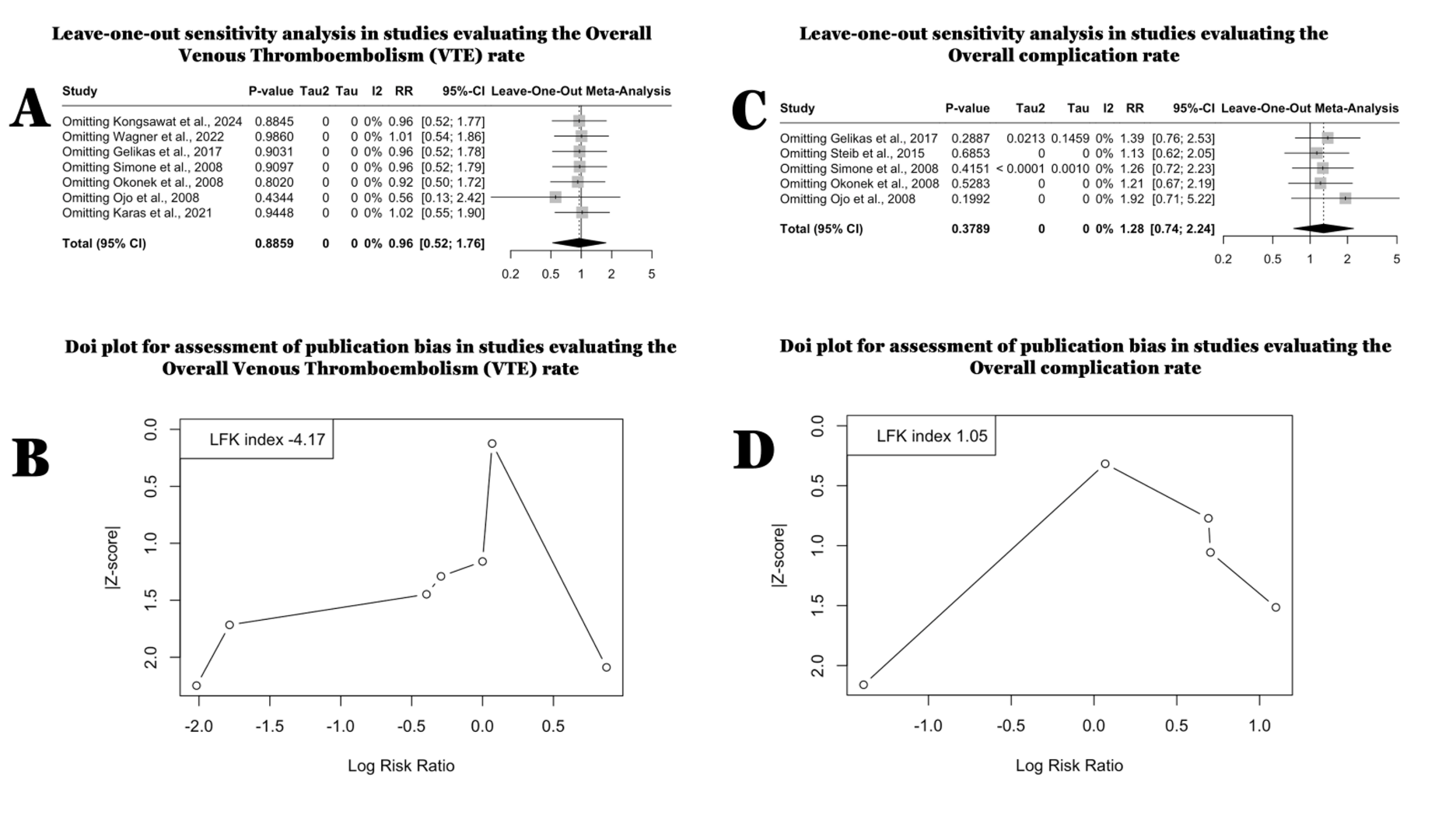
**

**(Fig. S2.5) Leave-one-out sensitivity analysis and Doi plot evaluating potential publication bias for VTE and overall complication rates.**

**
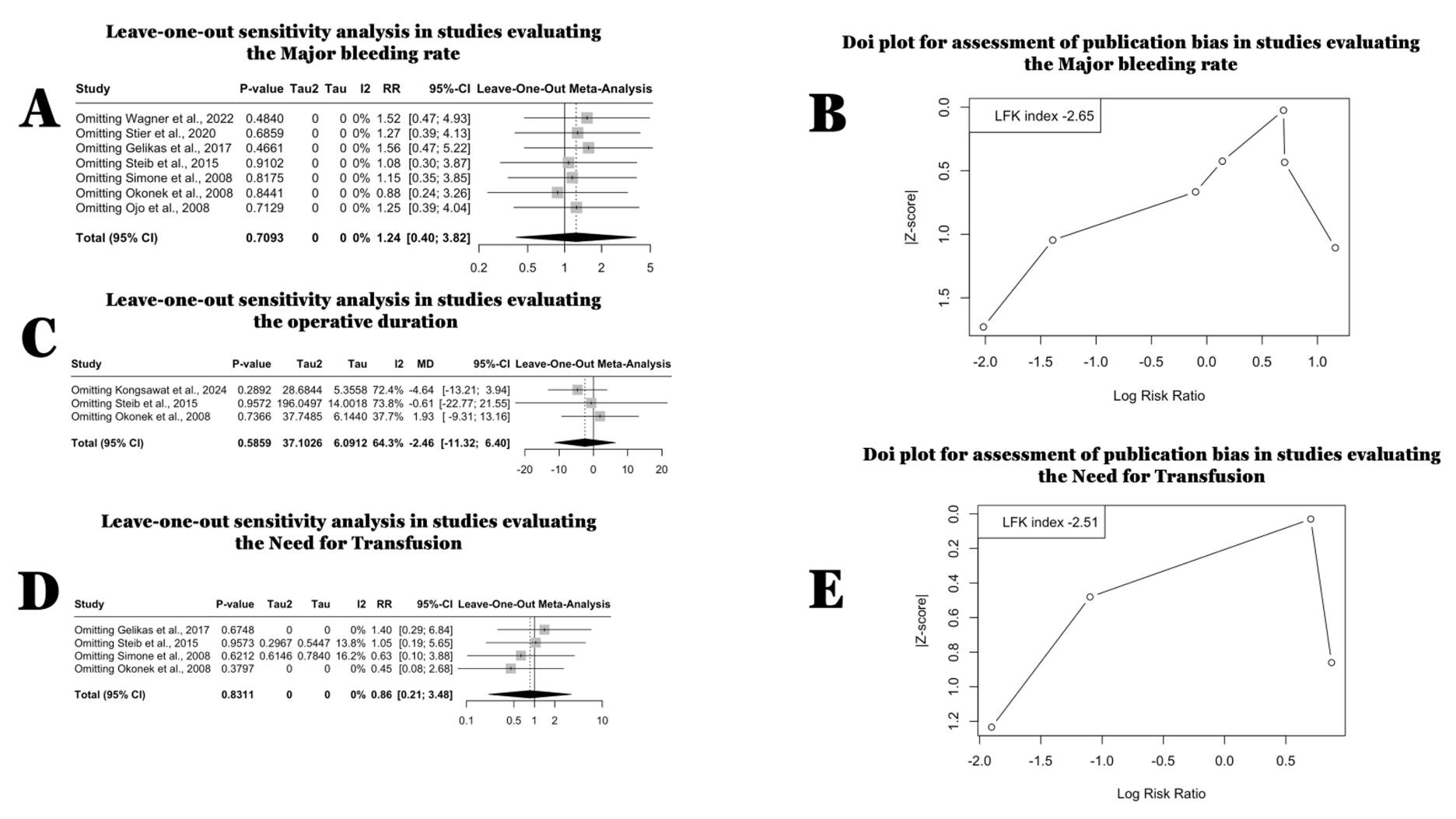
**

**(Fig. S2.6) Leave-one-out sensitivity analysis and Doi plot assessing potential publication bias regarding rates of major bleeding and transfusion needs, as well as the mean difference in operative duration.**
